# Multicomponent Synthesis of 4-Aryl-4,9-dihydro-1*H*-pyrazolo[3,4-*b*]quinolines Using L-Proline as a Catalyst—Does It Really Proceed?

**DOI:** 10.3390/molecules28227612

**Published:** 2023-11-15

**Authors:** Andrzej Danel, Elżbieta Porębska, Kacper Markiel, Oleksii Havrysh, Mateusz Kucharek, Arkadiusz Gut, Tomasz Uchacz

**Affiliations:** 1 The Faculty of Materials Engineering and Physics, Krakow University of Technology, Podchorążych Street 1, 30-084 Krakow, Poland; elzbieta.porebska@student.pk.edu.pl (E.P.); kacper.markiel@student.pk.edu.pl (K.M.); oleksii.havrysh@student.pk.edu.pl (O.H.); 2 The Faculty of Food Technology, Agricultural University, Balicka Street 122, 30-149 Krakow, Poland; mateusz.kucharek@urk.edu.pl; 3 The Faculty of Chemistry, Jagiellonian University, Gronostajowa Street 2, 30-387 Krakow, Poland; arkadiusz.gut@uj.edu.pl (A.G.); uchacz@chemia.uj.edu.pl (T.U.)

**Keywords:** 4-aryl-4,9*H*-3-methyl-1-phenyl-1*H*-pyrazolo[3,4-*b*]quinoline, multi-component reactions, L-proline, 4,4-(phenyl-methylene)-bis-(3-methyl-1-phenylpyrazol-5-oles)

## Abstract

Looking for effective synthetic methods for 1*H*-pyrazolo[3,4-*b*]quinolines preparation, we came across a procedure where, in a three-component reaction catalysed by L-proline, 4-aryl-4,9-dihydro-1*H*-pyrazolo[3,4-*b*]quinolines are formed. These compounds can be easily oxidised to a fully aromatic system, which gives hope for a synthetic method that could replace, e.g., Friedländer condensation, often used for this purpose, even though severely limited by the availability of suitable substrates. However, after careful repetition of the procedures described in the publication, it turned out that the compounds described therein do not form at all. The actual compounds turned out to be 4,4-(phenyl-methylene)-bis-(3-methyl-1-phenylpyrazol-5-oles). Therefore, 4-Aryl-4,9-dihydro-1*H*-pyrazolo[3,4-*b*]quinolines were prepared by another method and used as standards to compare the products formed in the original procedure.

## 1. Introduction

**Scheme 1:**
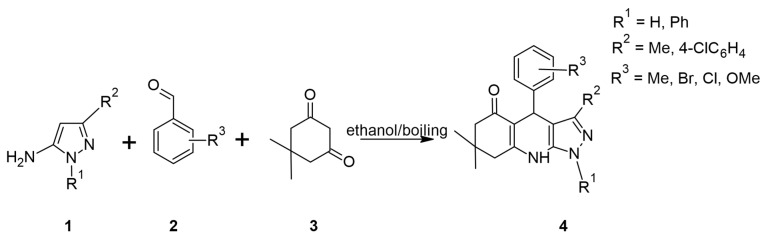
The first three-component synthesis of 1*H*-pyrazolo[3,4-*b*]quinolines.

In 1901, Willstätter synthesised tropinone, a precursor to the tropane alkaloid atropine. This compound was obtained by a 21-step synthesis from cyclopentanone with an overall yield of 0.75% [1]. The opposite of this synthesis is shown in the work published in 1917 by Robinson, who obtained the mentioned tropinone in a one-step, three-component reaction with a yield of 17% [2]. This synthesis is one of the pioneering multi-component chemical reactions. Earlier reactions of this type include Strecker’s synthesis of amino acids from aldehydes, NH_3_ and HCN published in 1854, or the slightly later Mannich reaction [3,4]. The subject of multi-component syntheses has been discussed in numerous reviews and is also presented in many monographs [5,6,7,8]. The 1*H*-pyrazolo[3,4-*b*]quinoline, which is the focus of the research in the current publication, can also be obtained using multi-component reactions. Only some of these will be mentioned, because an exhaustive discussion of this topic was made in our recent review [9]. The first reaction of this kind was described in 1998 by Hormanza et al. (Scheme 1) [10].

**Scheme 2:**
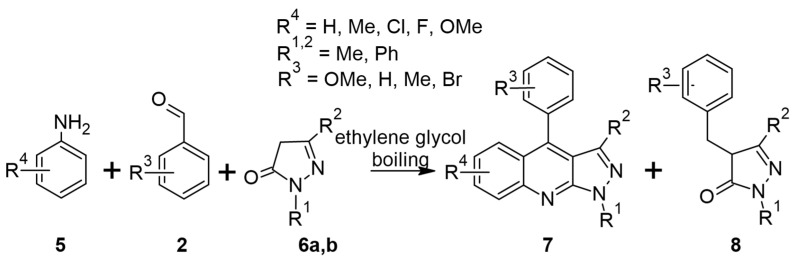
The three-component synthesis of fully aromatic 1*H*-pyrazolo[3,4-*b*]quinolines.

They used 5-aminopyrazole **1**, aromatic aldehyde **2**, and dimedone **3** for this purpose. During boiling in ethanol, the formation of the corresponding 1*H*-pyrazolo[3,4-*b*]quinoline **4** was observed, in which both the carbocyclic and the middle rings were not aromatic. Another important reaction leading to 4-aryl-1*H*-pyrazolo[3,4-*b*]quinolines **7** is the procedure described by Tomasik et al. [11]. They reacted substituted anilines **5**, aromatic aldehydes **2** and 2,5-diphenyl-2,4-dihydro-3*H*-pyrazol-5-one (**6a**, R^1,3^ = Ph) or 2-phenyl-5-methyl-2,4-dihydro-3*H*-pyrazol-3-one (**6b**, R^1^ = Ph, R^2^ = Me) (Scheme 2).

**Scheme 3:**
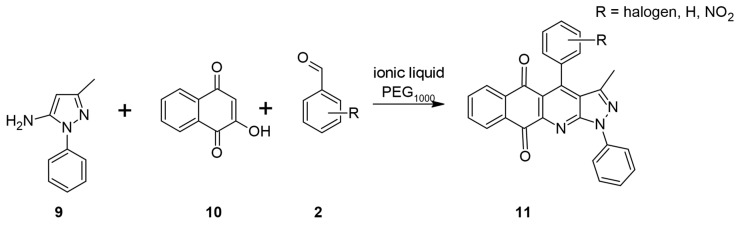
The three-component synthesis of 1*H*-pyrazolo[3,4-*b*]quinolines with aromatic pyridine moiety.

In this case a fully aromatic product **7** is obtained and benzal derivative **8** was also isolated. The reaction yields of **7** ranged from 2–33%. At present, this is the only example of this type of multi-component reaction where a 1*H*-pyrazolo[3,4-*b*]quinoline **7** system is formed. In other cases, compounds with an aromatic pyridine ring and a saturated carbocyclic system are obtained. An example of such a reaction is shown in Scheme 3, where 5-amino-3-methyl-1-phenylpyrazole **9**, 2-hydroxy-1,4-naphthalenedione **10** and aromatic aldehyde **2** were used. The reaction was carried out in the presence of an ionic liquid and PEG_1000_ and led to the formation of derivatives **11** [12,13].

**Scheme 4:**
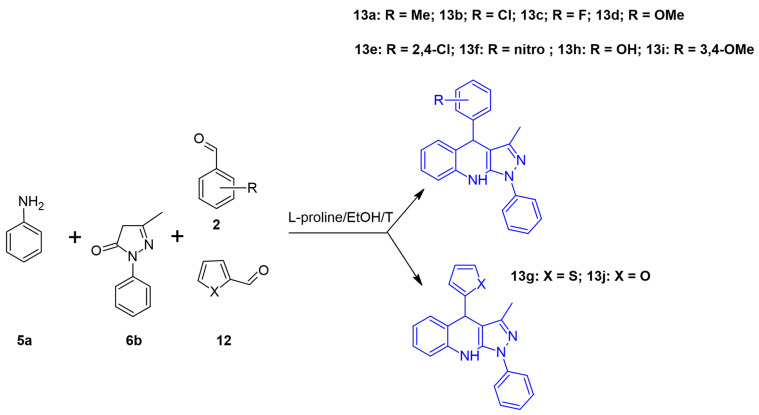
The potential synthesis of 4-aryl-4,9-dihydro-1*H*-pyrazolo[3,4-*b*]quinolines **13**.

In recent years, many multi-component reactions using L-proline as a catalyst have been mentioned for the synthesis of heterocyclic systems [14,15,16,17]. In 2017, Hegde and Shetty used aniline **5a**, aromatic aldehyde **2/12**, 2-phenyl-5-methyl-2,4-dihydro-3*H*-pyrazol-3-one **6b** and L-proline as a catalyst in a reaction leading to pyrazolo[3,4-*b*]quinolines **13** (Scheme 4) [18].

They obtained a series of eight 4-aryl-4,9-dihydro-3-methyl-1-phenyl-1*H*-pyrazolo[3,4-*b*]quinolines **13a–f, h, i** with an aromatic carbocyclic ring and two with a heterocyclic **13g,j** in the 4-position, which has not been found in the reactions reported so far. The final yields were relatively high, reaching 78–89%.

## 2. Results and Discussion

**Scheme 5:**
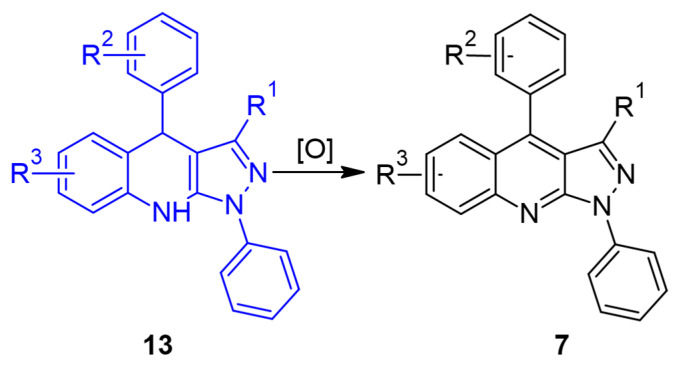
Potential oxidative aromatisation of 4-aryl-4,9-dihydro-1*H*-pyrazolo[3,4-*b*]quinolines.

The reactions published in the paper by Hedge and Shetty seemed very interesting because the obtained compounds **13** can be oxidised to give fully aromatic systems **7** (Scheme 5).

**Scheme 6:**
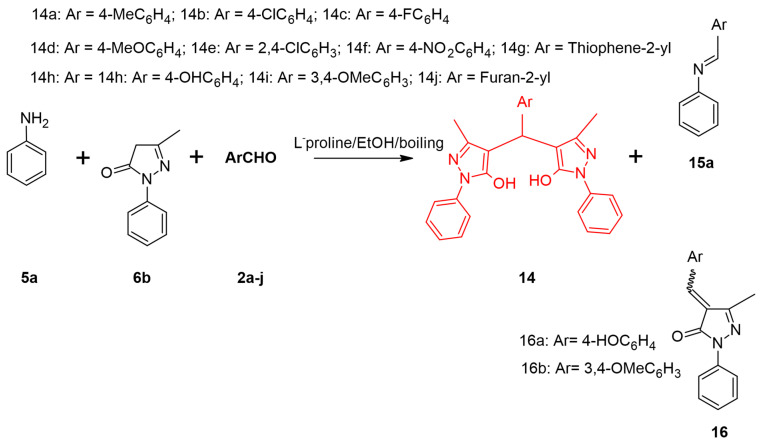
The synthesis of 4,4′-(aryl-methylene)-bis-(3-methyl-1-phenylpyrazol-5-oles) **14**.

An example of such a reaction is the oxidation of pyrazolines to pyrazoles, which can be performed, e.g., with oxygen in acetic acid, MnO_2_, DDQ or other oxidants [19,20,21]. Moreover, it can be expected that the overall yield could be greater than 33%, which is a serious shortcoming of the previous reaction described by Tomasik et al. For this reason, the reactions presented in Scheme 4 were very interesting and we decided to use their potential when it comes to the synthesis of fully aromatic 1*H*-pyrazolo[3,4-*b*]quinolines [22,23]. At first, we decided to test some of the procedures described by Hegde and Shetty and *p*-tolu-aldehyde **2a**, *p*-chlorobenzaldehyde **2b** and 2,4-dichlorobenzaldehyde **2e** were selected for the experiments. We chose these three compounds, among others, because the authors included the spectra data of resulted 4,9-dihydro-1*H*-pyrazolo[3,4-*b*]quinolines **13a,b,e** in Supplementary Materials in the form of graphics. In the original publication, the reaction was carried out on a scale of 1 mmol, which we increased to 5 mmol to facilitate the isolation of the product, which in most cases precipitated after a few minutes of heating. The yields resulted in three products that were really high, and standard ^1^H NMR and ^13^C NMR analyses were performed. Unfortunately, it turned out that the obtained compounds were not the expected 4-aryl-4,9-dihydro-1*H*-pyrazolo[3,4-*b*]quinoline **13a,b,e** derivatives at all. Instead, only the 4,4′-(aryl-methylene)-bis-(3-methyl-1-phenylpyrazol-5-oles **14a,b,e** were isolated (Scheme 6). The same situation happened with other aldehydes **2c,d,f,g**. When *p*-hydroxybenzaldehyde **2h** and 3,4-dimethoxybenzaldehyde **2i** were reacted under the conditions described by Hegde and Shetty the orange precipitates **16a** and **16b** were isolated as products. Increasing the amount of solvent led to the formation of appropriate 4,4′-(aryl-methylene)-bis-(3-methyl-1-phenylpyrazol-5-oles **14h** and **14i** again. When we used 2-furylaldehyde, we were unable to isolate any products from the post-reaction mixture due to its tarnation, even though we used freshly purified aldehyde. However **14j** was detected with TLC in the reaction mixture using an authentic sample prepared by another method. In addition, in the case of *p*-tolu-aldehyde, Schiff’s base **15** (Ar = C_6_H_4_Me) was isolated from the post-reaction mixture. The compound was identified by comparison with a separately synthesised Schiff base, prepared from aniline and *p*-tolu-aldehyde and with ^1^H NMR spectrum [24]. Appropriate Schiff bases were also detected with TLC in the post-reaction mixtures in the other two cases. Extending the reaction time from 5–6 h up to 24 h did not cause any changes as far as the final product was concerned. What is particularly surprising is that, when we removed L-proline from the reaction medium, we also obtained only compounds **14** and traces of Schiff bases **15**. So there is a clear conclusion that L-proline has no part in this reaction at all in spite of the fact that, in the original publication, the authors proposed a potential mechanism for this reaction, with an emphasis on the role of this amino acid.

To finally remove any doubts about the product obtained by Hegde and Shetty, we performed additional syntheses by heating the pyrazolone **6b** and a few aldehydes, namely **2a, 2i** and **2j,** in a 2:1 molar ratio. The obtained compounds **14** are known and described in the literature, hence there are no doubts about their structure. When we compared the R_f_ values of these products with compounds obtained in the three-component reaction with L-proline and without L-proline using TLC chromatography, it turned out that they were identical. 

Our results are in part supported by the work of Hennig and colleagues who studied the reactions of azomethines with CH-acids. Among the entire series of the latter, they tested the reaction of pyrazolone **6b,** aniline **5a** and aromatic aldehydes **2**. Depending on the reaction conditions, **14**, **16** and **15** were formed and the potential formation of **13** was not mentioned at all [25].

It is worth considering on what basis the authors assigned the structure of **13** to compounds **14**. In our opinion, there was an incorrect interpretation of the ^1^H NMR spectra of **13a-j** (Scheme 4). If we analyse these spectra included in the publication they have a common element, namely peaks in the range of 14.00–13.00 ppm and occasionally at 5 ppm. The authors attribute the first of these to the proton bounded to nitrogen in position 9 of **13** and the second to the methine proton at carbon C-4 of the parent skeleton. The collected data are included in the first and second column (Table 1).

In the table we have included the value of ^1^H NMR shifts of the protons associated with oxygen and the methine protons of **14** (the third and fourth column), which correspond to the alleged **13** obtained by Hedge and Shetty, collected on the basis of some literature resources [27,29,30]. An example of such a publication is the work of Mohammadi and Ghorbani-Choghamarani, who synthesised **14** (R = 4-Me, 4-Cl) using magnetic nanocomposites modified with sulfone groups [26]. If we take into account the ^1^H NMR shift values of protons attached to nitrogen in position 9 for *p*-tolu-benzaldehyde and *p*-chlorobenzaldehyde derivatives **13** in the Hedge and Shetty paper, they are 14.00 and 13.90 ppm, respectively [18]. The corresponding shift values of the methine protons at the C-4 position are 4.89 ppm and 4.96 ppm. When we compare these values with the data published in the Mohammadi and Ghorani-Choghamarani paper, we notice that they are almost identical, except that the values at the 13.94 ppm and 13.89 ppm correspond to protons attached to oxygen atoms of **14** and not nitrogen N-9 atoms in **13**. In the ^1^H NMR spectra included in the work by Hegde and Shetty, one can also notice very weak broad peaks in the region of 12–13 ppm, which are identical to the peaks at 12.44 and 12.53 ppm coming from hydrogens associated with oxygen. In some derivatives **14,** these peaks are not always visible.

**Figure 1:**
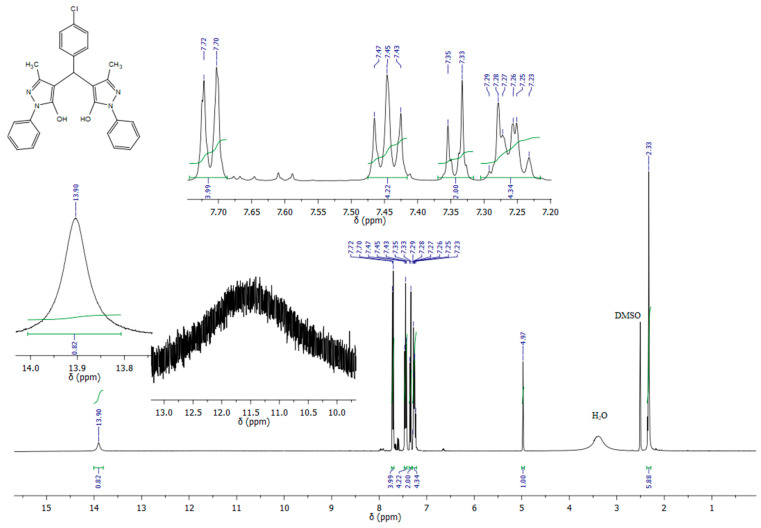
^1^H NMR spectra of 4,4′-(4-chlorophenylmethylene)-bis-(3-methyl-1-phenylpyrazol-5-ol) **14b**.

In Figure 1 we included a ^1^H NMR spectrum of 4,4′-(4-chlorophenylmethylene)-bis-(3-methyl-1-phenyl-pyrazol-5-ole) **14b** and these two peaks can be seen.

In the case of the derivative obtained from *p*-chlorobenzaldehyde, the authors included the measurement result of a mass spectrum (ESI) of the sample, obtaining values for [M^+^] 371 (100%), which corresponds to the structure of 4,9-dihydro-1-*H*-pyrazolo[3,4-*b*]quinoline **13b** (R = Cl). Elemental analysis also confirms this structure. However, the ^1^H NMR spectrum indicates that in fact product **14b** was formed. Unfortunately, we do not know where this inaccuracy came from. 

**Figure 2:**
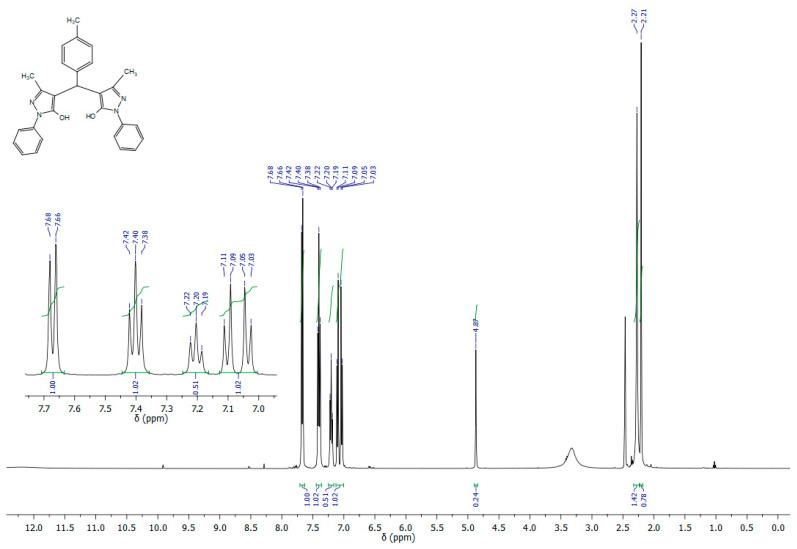
^1^H NMR spectra of 4,4′-(4-methylphenylmethylene)-bis-(3-methyl-1-phenylpyrazol-5-ol) **14a**.

Another problem occurs in the case of a product obtained from *p*-tolu-aldehyde **2a**. The ^1^H NMR spectra of our compound is depicted in Figure 2.

In the case of the ^1^H NMR spectrum of alleged 4-(*p*-methylphenyl)-4,9-dihydro-3-methyl-1-phenyl-1*H*-pyrazolo[3,4-*b*]quinoline **13a** (R = Me), the authors assigned (in their publication) each of the peaks located at 2.30 and 2.24 ppm, respectively, to three protons from the methyl groups in each case. In fact, if we look closer at the ^1^H NMR spectrum for **14a**, the ratio of the number of protons at 2.27 and 2.21 is 2:1, so it cannot be the compound **13a** at all. The spectra from the publication of Hedge and Shetty are identical to those we obtained and the analysed compound cannot be 4-(*p*-methylphenyl)-1-phenyl-3-methyl-4,9-dihydro-1*H*-pyrazolo[3,4-*b*]quinoline **13a** (Scheme 4), though elemental analysis again confirmed the molecular structure of **13a**. 

Based on our results, we can conclude that the three-component reaction of aniline **5a**, aromatic aldehydes **2a-j**, 2-phenyl-5-methyl-2,4-dihydro-3*H*-pyrazol-3-one **6b** and a catalytic amount of L-proline is completely useless when it comes to the synthesis of 4-aryl-4,9-dihydro-1*H*-pyrazolo[3,4-*b*]quinolines **13a-j** (Scheme 4). 

**Scheme 7:**
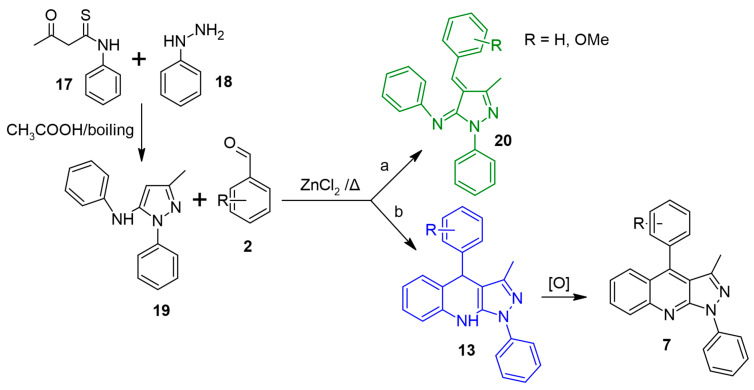
Cyclisation of 5-*N*-phenyl-3-methyl-1-phenylpyrazole to 4-aryl-4,9-dihydro-1*H*-pyrazolo[3,4-*b*]quinolines.

In such a case, the question arises whether the previously mentioned structures **13** can be synthesised. A different approach was used to check this. In 1911, Michaelis described the synthesis of 4-benzylidene-5-*N*-phenylaminopyrazoles **20** by reacting aromatic aldehydes **2** with 5-*N*-phenyl-3-methyl-1-phenylpyrazole **19** (Scheme 7) [31]. The author reported in the publication that he obtained two derivatives of this kind. However, later research by Tomasik et al. showed that the actual products formed in this reaction were 1*H*-pyrazolo[3,4-*b*]quinolines **7** [32].

**Scheme 8:**
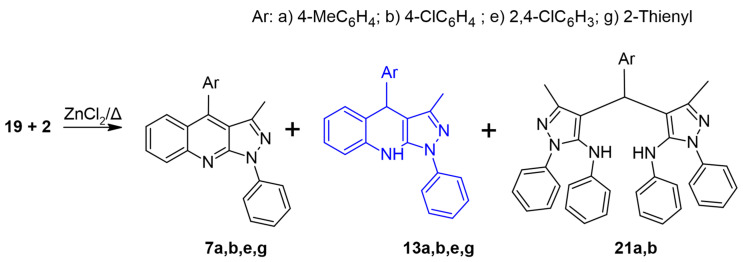
The final products from the Michaelis protocol.

Michaelis and the authors of the later publication did not study the course of the reaction, but it can be expected that, during it, intermediate products **13** can be formed and correspond to those described in the work of Hedge and Shetty. To test this hypothesis, the appropriate amino-pyrazole **19** was synthesised from phenyl-hydrazine **18** and 3-oxo-*N*-phenylbutanethioamide **17**. Next, it was reacted with aldehydes **2a**, **2b**, **2e** and **2g** in the presence of anhydrous ZnCl_2_. Michaelis and Tomasik and their co-workers carried out a melting reaction of aromatic aldehyde **2** with 5-*N*-phenylpyrazole **19** in an open flask with the final yields of **7** in the order of 40–50%. This was slightly larger when using a microwave field. If it is assumed that the intermediate product in this reaction is the 4,9-dihydro-1*H*-pyrazolo[3,4-*b*]quinoline **13**, it can be taken for granted that the oxygen present in the air plays a significant role in the oxidation of this compound. In order to eliminate its influence, the reaction was carried out in closed ampoules for 6 h. At the end of heating and analysis of the post-reaction mixture by TLC, the presence of three products was found, namely 4-aryl-1*H*-pyrazolo[3,4-*b*]quinoline **7a,b,e,g,** 4-aryl-4,9-dihydro-1*H*-pyrazolo[3,4-*b*]quinoline **13a,b,** and 4,4′-(arylmethylene)-bis-[3-methyl-*N*,1-diphenyl-1*H*-pyrazol-5-amine] **21a,b** (Scheme 8).

The yield of 1*H*-pyrazolo[3,4-*b*]quinoline was definitely lower (5–8%) than in the case of the reaction carried out under air condensation (as in the original work by Michaelis). Separation of the products was very troublesome due to the almost identical starting values R_f_ **19** and resulted in 4,9-dihydro-1*H*-pyrazolo[3,4-*b*]quinolines **13**. The products were separated by pre-removal of 1*H*-pyrazolo[3,4-*b*]quinolines **7** with residual unreacted aldehyde after digesting of the reaction mixture with methanol, in which pyrazolo-quinolines are insoluble. The filtrate was subjected to column chromatography using a mixture of toluene and ethyl acetate in a ratio of 3:0.1 with a gradual increase in the amount of ethyl acetate to 3:0.4 to separate off unreacted **19, 13** and **21**.

**Figure 3:**
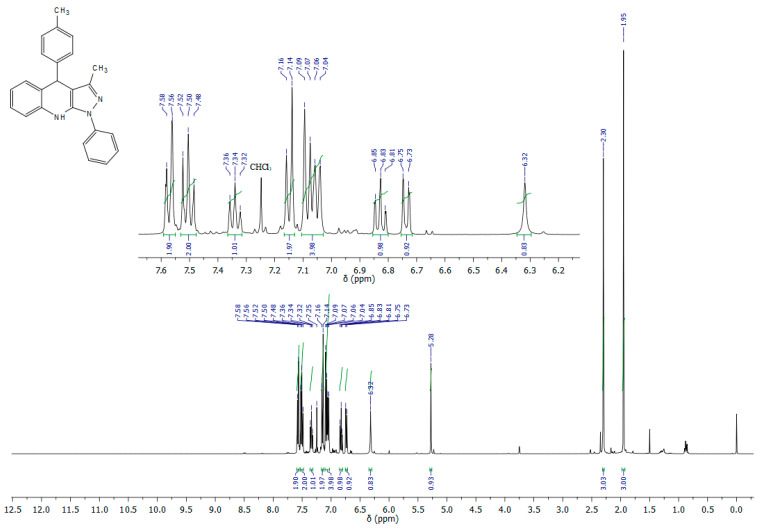
^1^H NMR spectra of 4-(4-methylphenyl)-4,9-dihydro-3-methyl-1-phenyl-1*H*-pyrazolo[3,4-*b*]quinoline **13a**.

**Figure 4:**
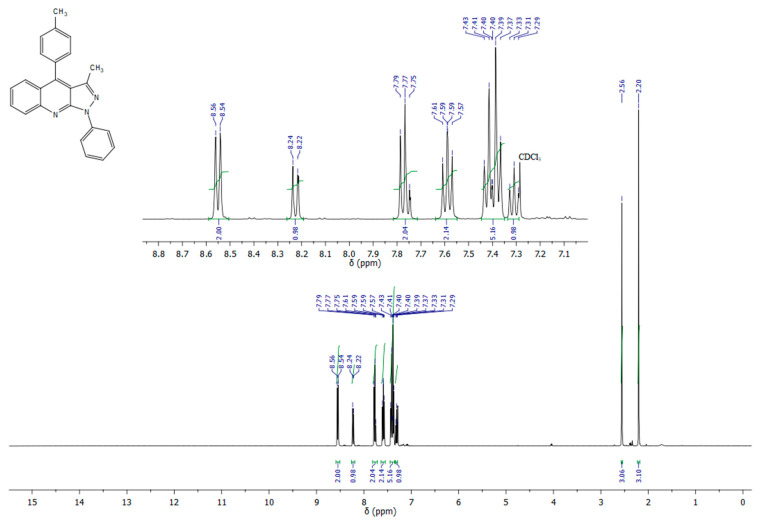
^1^H NMR spectrum of 4-(4-methylphenyl)-3-methyl-1-phenyl-1*H*-pyrazolo[3,4-*b*]quinoline **7a**.

We performed the reaction on only four aldehydes **2a,b,e,g** because, in the case of the others, we were not able to separate the final products **13** and **21** with a sufficient purity for analysis. In case of furfural **2j,** the only result we received was a black tar. Figure 3 shows the ^1^H NMR spectrum of 4-(4-methylphenyl)-1-phenyl-3-methyl-4,9-dihydro-1*H*-pyrazolo[3,4-*b*]quinoline **13a**. The signals presented in the spectrum do not coincide at all with those that are included in the Supplementary Materials of Shetty and Gatta. The proton at N-9 is visible at 6.32 ppm instead of 14.00 ppm (compare with Table 1). Both protons at 6.32 ppm and 5.28 ppm disappear after oxidation of **13a** when 1*H*-pyrazolo[3,4-*b*]quinoline **7a** is formed (Figure 4).

After obtaining and establishing the structure, compounds **13a**, **13b, 13e** and **13g** were used as TLC standards when the three-component reaction was repeated with L-proline as a catalyst. These products were not found even in trace amounts. Taking into account the experiments performed and the interpretation of the results, the issue of the synthesis of pure 4-aryl-4,9-dihydro-1*H*-pyrazolo[3,4-*b*]quinolines using a multi-component reaction remains a challenge.

## 3. Materials and Methods

### 3.1. Chemicals and Instruments

Chemicals and solvent were purchased from Aldrich/Merck and POCh (Polish chemical company) respectively. ^1^H NMR and ^13^C NMR spectra were recorded using the Bruker Advance III (400 MHz) and the Bruker Advance III (600 MHz) spectrometers (Jagiellonian University, Faculty of Chemistry, Kraków, Poland). Elemental analysis was performed using the CHNS Vario MICRO Cube analyser with electronic micro-balance. Melting points were measured using a MEL-TEMP II cryometer (Agricultural University, Faculty of Food Chemistry). TLC chromatograms were visualised using a dual-band (254 and 365 nm) Spectroline UV lamp model ENF-260/FE and 70–230 mesh ASTM silica gel purchased from Merck KGaA (Darmstadt, Germany) was used as the stationary phase in the column chromatographic methods. The 70–230 mesh ASTM (Activity Grade I) alumina was also purchased at Merck KGaA. The mass spectra were taken with a mass spectrometer coupled to a Shimadzu LCMS-8040 high performance liquid chromatograph (Jagiellonian University, Faculty of Chemistry).

### 3.2. Experimental Procedures

#### 3.2.1. An Attempt at the Synthesis of 4-Aryl-1-phenyl-3-methyl-4,9-dihydro-1*H*-pyrazolo[3,4-*b*]quinolines via a Three-Component Reaction According to the Hegde and Shetty Protocol

Original Procedure by Hegde and Shetty [18]

“L-Proline (15 mg, 0.2 mmol) was added to a mixture of 5-methyl-2-phenyl-2,4-dihydro-3*H*-pyra zol-3-one (174 mg, 1 mmol), aryl aldehyde (1 mmol) and aniline (91 mg, 1 mmol) in EtOH (2 mL), and the solution was refluxed for 5–6 h. The progress of the reaction was monitored by TLC (eluent hexane—EtOAc, 2:1). After completion of the reaction, the mixture was poured into ice-cold water. The solid was filtered off, washed with EtOH, and recrystallised from EtOAc.”

Procedure (a) 865 mg (5 mmol) of 2-phenyl-5-methyl-2,4-dihydro-3*H*-pyrazol-3-one, 600 mg (5 mmol) *p*-tolu-aldehyde, 450 mg (5 mmol) aniline, 75 mg (20 mol%) L-proline and 10 mL ethanol were introduced into a round bottom flask (25 mL) and placed in a heating block and boiled for 5 h under a reflux condenser. After heating, the reaction mixture was placed in a refrigerator and left for 24 h; the separated crystalline precipitate was then filtered off.

Procedure (b): 5 mmoles of substrates without L-proline were used in the reaction.

4,4′-(4-Methylphenylmethylene)-bis-(3-methyl-1-phenylpyrazol-5-ol) **14a**.

Procedure a: Colourless crystals, 820 mg, yield 72%, mp. 204–205 °C. Lit. 203–205 °C [28].

Procedure b: Colourless crystals, 880 mg, yield 78%, mp. 204–205 °C.

^1^H NMR (400 MHz, DMSO-d_6_,) δ, ppm (*J*, Hz): 7.67 (d, *J* = 7.7 Hz, 4H), 7.40 (t, *J* = 7.9 Hz, 4H), 7.20 (t, *J* = 7.3 Hz, 2H), 7.10 (d, *J* = 8.1 Hz, 2H), 7.04 (d, *J* = 8.1 Hz, 2H), 4.87 (s, 1H); 2.27 (s, 6H), 2.21 (s, 3H) [26]. ^13^C NMR (101 MHz, DMSO-d_6_) δ, ppm: 146.8, 139.7, 135.3, 129.5, 129.2, 127.6, 126.1, 121.0, 33.3, 21.1, 12.1.

4-Methyl-*N*-[(1*E*)-phenyl-methylidene]aniline **15a**.

The filtrate was evaporated, and the resulting oil was dissolved in chloroform and dried with anhydrous MgSO_4_. The chloroform was evaporated and the oil obtained was dissolved in petroleum ether (40/60) and chromatographed on a silica gel column. The solution was evaporated and the light-yellow oil was left in the refrigerator to solidify. Pale yellow crystalline mass, 173 mg, yield 18%, mp. 40–42 °C. Lit. 40–41°C [24]. TLC testing (toluene:petroleum ether 40/60 ratio 1:1) showed the sample to have identical R_f_ values to the original Schiff base synthesised from aniline and *p*-tolu-aldehyde.

^1^H NMR (400 MHz, CDCl_3_) δ, ppm (*J*, Hz): 8.31 (s, 1H, -N=C**H**-); 7.69 (d, J = 8.1 Hz, 2H); 7.31–7.26 (m, 2H); 7.17 (d, J = 7.9 Hz, 2H); 7.14–7.07 (m, 3H); 2.31 (s, 3H, -CH_3_). ^13^C NMR (101 MHz, CDCl_3_) δ, ppm: 160.38; 152.31; 141.89; 133.73; 129.46; 129.17; 128.87; 125.80; 120.94; 21.80.

4,4′-(4-Chlorophenylmethylene)-bis-(3-methyl-1-phenylpyrazol-5-ol) **14b**

Procedure a: Colourless crystals, 878 mg, 74%, mp. 205–206 °C. Lit. 206–208 °C [33]

Procedure b: Colourless crystals, 950 mg, 80%, mp. 205–206 °C.

^1^H NMR (400 MHz, CDCl_3_) δ, ppm (*J*, Hz): 13.90 (s, 1H), 7.71 (d, *J* = 7.6 Hz, 4H), 7.45 (t, *J* = 8.0 Hz, 4H), 7.34 (d, *J* = 8.6 Hz, 2H), 7.30–7.21 (m, 4H), 4.97 (s, 1H), 2.33 (s, 6H) [26]. ^13^C NMR (100 MHz, CDCl_3_) δ, ppm (*J*, Hz): 146.68, 141.63, 131.01, 129.59, 129.42, 129.38, 128.47, 121.02, 33.02, 12.06.

4,4′-(4-Fluorophenylmethylene)-bis-(3-methyl-1-phenylpyrazol-5-ol) **14c**.

Procedure a: Colourless crystals, 900 mg, 79%, mp. 168–169 °C. Lit. 180–181 °C [33].

Procedure b: Colourless crystals, 980 mg, 86%, mp. 167–168 °C.

^1^H NMR (400 MHz, DMSO-d_6_) δ, ppm (*J*, Hz): 13.95 (s, 1H), 7.72 (d, *J* = 7.7 Hz, 4H), 7.45 (t, *J* = 7.9 Hz, 4H), 7.31–7.23 (m, 4H), 7.11 (t, *J* = 8.9 Hz, 4H), 4.97 (s, 1H), 2.33 (s, 6H) [27].

4,4′-(4-Methoxyphenylmethylene)-bis-(3-methyl-1-phenylpyrazol-5-ol) **14d**.

Procedure a: Colourless crystals, 230 mg (2 mmol scale), yield 49%, mp. 167–169 °C. Lit. 165–167 °C [33].

^1^HNMR (400 MHz, DMSO-d_6_) δ, ppm (*J*, Hz): 7.67 (d, *J* = 7.9 Hz, 4H), 7.40 (t, *J* = 7.6 Hz, 4H), 7.20 (t, *J* = 7.0 Hz, 2H), 7.12 (d, *J* = 8.3 Hz, 2H), 6.80 (d, *J* = 8.4 Hz, 2H), 4.86 (s, 1H), 4.86 (s, 3H), 2.27 (s, 6H) [27]. ^13^C NMR (100 MHz, DMSO-d_6_) δ, ppm (*J*, Hz): 158.04, 146.69, 134.45, 129.28, 128.70, 126.03, 121.03, 114.03, 55.52, 32.87, 11.73.

4,4′-(2,4-Dichlorophenylmethylene)-bis-(3-methyl-1-phenylpyrazol-5-ol) **14e**

Procedure a: Colourless crystals, 1100 mg, yield 86%, p. 234–235 °C. Lit. 228–230 °C [32].

Procedure b: Colourless crystals, 1000 mg, yield 79%, p. 233–235 °C.

^1^H NMR (400 MHz, DMSO-d_6_,) δ, ppm (*J*, Hz): 13.83 (s, 1H), 7.76 (d, *J* = 8.5 Hz, 1H), 7.70 (d, *J* = 7.7 Hz, 4H), 7.55 (d, *J* = 2.2 Hz, 1H, 7.43 (dt, *J* = 8.9, 5.0 Hz, 5H 7.25 (t, *J* = 7.4 Hz, 2H), 5.10 (s, 1H), 2.29 (s, 6H) [28]. ^13^C NMR (100 MHz, DMSO-d_6_,) δ, ppm: 146.47, 138.93, 133.36, 132.12, 131.90, 129.39, 129.31, 127.45, 126.15, 121.07, 31.84, 12.28.

4,4′-(4-Nitrophenylmethylene)-bis-(3-methyl-1-phenylpyrazol-5-ol) **14f**.

Procedure a: Colourless crystals, 850 mg, yield 71%, mp. 234–235 °C. Lit. 228–230 °C [32].

Procedure b: Colourless crystals, 900 mg, 75%, mp. 233–234 °C.

^1^H NMR (400 MHz, DMSO-d_6_,) δ, ppm (*J*, Hz): 13.87 (s, 1H), 8.18 (d, *J* = 8.9 Hz, 2H), 7.71 (d, *J* = 7.6 Hz, 4H), 7.53 (d, *J* = 8.4 Hz, 2H), 7.45 (t, *J* = 8.0 Hz, 4H), 7.26 (t, *J* = 7.4 Hz, 2H), 5.14 (s, 1H), 2.36 (s, 6H) [28].^13^C NMR (101 MHz, DMSO-d_6_,) δ, ppm: 150.78, 146.75, 146.38, 129.40, 129.08, 126.20, 123.80, 121.07, 33.64, 12.05.

4,4′-(2-Thienylmethylene)-bis-(3-methyl-1-phenylpyrazol-5-ol) **14g**.

Procedure a: Colourless crystals, 140 mg (2 mmol scale), 33%, mp. 187–189 °C. Lit. 189–190 °C [34].

Procedure b: Colourless crystals, 500 mg (5 mmol scale), 45%, mp. 187–188 °C.

^1^H NMR (400 MHz, DMSO-d_6_,) δ, ppm (*J*, Hz): 7.67 (d, *J* = 8.6 Hz, 1.0 Hz, 4H), 7.41 (t, *J* = 8.0 Hz, 4H), 7.27–7.18 (m, 3H), 6.87 (dd, *J* = 5.1 Hz, 3.5 Hz, 1H), 6.73–6.69 (m, 1H), 5.09 (s, 1H), 2.28 (s, 6H) [26]. ^13^C NMR (101 MHz, DMSO-d_6_) δ, ppm: 148.06, 146.34, 129.48, 127.30, 126.22, 124.69, 124.59, 29.98, 12.05.

4,4′-(4-Hydroxyphenylmethylene)-bis-(3-methyl-1-phenylpyrazol-5-ol) **14h**.

The reaction was carried out on a 5 mmol scale using 30 mL of ethanol as a solvent.

Procedure a: Yellow crystals, 890 mg, yield 78%, mp. 158–160 °C. Lit. 153–155 °C [30].

Procedure b: Yellow crystals, 930 mg, yield 82%, mp. 158–159 °C.

^1^H NMR (400 MHz, DMSO-d_6_,) δ, ppm (*J*, Hz): 13.94 (s, 1H), 9.18 (s, 1H), 7.72 (d, *J* = 7.8 Hz, 4H), 7.45 (t, *J* = 7.9 Hz, 4H), 7.25 (t, *J* = 7.3 Hz, 2H), 7.06 (d, *J* = 8.5 Hz, 2H), 6.68 (d, *J* = 8.6 Hz, 2H), 4.86 (s, 1H), 2.31 (s, 6H) [30]. ^13^C NMR (101 MHz, DMSO-d_6_) δ, ppm: 155.96, 146.64, 132.73, 129.36, 128.55, 125.96, 120.95, 115.31, 32.84, 12.09.

4,4′-(3,4-Dimethoxyphenylmethylene)-bis-(3-methyl-1-phenylpyrazol-5-ol) **14i**.

The reaction was carried out on a 5 mmol scale using 30 mL of ethanol as a solvent.

Procedure a: Colourless crystals, 1050 mg, 85%, mp. 208–209 °C. Lit. 194–196 °C [30].

Procedure b: Colourless crystals, 1100 mg, 89%, mp. 207–209 °C.

^1^H NMR (400 MHz, DMSO-d_6_,) δ, ppm (*J*, Hz): 14.02 (s, 1H), 12.39 (s, 1H), 7.71 (d, *J* = 7.7 Hz, 4H), 7.45 (t, *J* = 7.9 Hz, 4H), 7.25 (t, *J* = 7.3 Hz, 2H), 6.85 (ddd, *J* = 17.9, 8.6, 1.6 Hz, 3H), 4.89 (s, 1H), 3.71 (s, 1H), 3.66 (s, 1H), 2.32 (s, 6H) [29]. ^13^C NMR (101 MHz, DMSO-d_6_) δ, ppm: 148.86, 147.70, 146.63, 135.42, 129.38, 121.05, 119.76, 112.23, 112.09, 56.02, 55.96, 33.38, 12.13.

4,4′-(2-Furylmethylene)-bis-(3-methyl-1-phenylpyrazol-5-ol) **14j**.

The compound could not be isolated from the post-reaction mixture, but its presence was detected using TLC and compared with the original substance obtained in the reaction of pyrazolone **6b** and 2-furylaldehyde (Section 3.2.2. **14j**).

(4*E*)-4-[(4-hydroxyphenyl)methylidene]-5-methyl-2-phenyl-2,4-dihydro-3*H*-pyrazol-3-one **16a**

Procedure a: Orange crystals, 970 mg, yield 69%, mp. 239–240 °C. Lit. 238 °C [35]

^1^H NMR (600 MHz, DMSO-d_6_) δ, ppm (*J*, Hz): 10.84 (s, 1H), 8.64 (d, *J* = 8.8 Hz, 2H), 7.93 (d, *J* = 7.6 Hz, 2H), 7.70 (s, 1H), 7.45–7.40 (m, 2H), 7.18 (t, *J* = 7.4 Hz, 1H), 6.95 (d, *J* = 8.9 Hz, 2H), 2.32 (s, 3H).

(4*E*)-4-[(3,4-dimethoxyphenyl)methylidene]-5-methyl-2-phenyl-2,4-dihydro-3*H*-pyrazol-3-one **16b**.

Procedure a: Orange crystals, 1230 mg, yield 76%, mp. 165–166 °C.

^1^H NMR (600 MHz, CDCl_3_) δ, ppm (*J*, Hz): 9.02 (s, 1H), 7.97 (d, *J* = 7.6 Hz, 2H), 7.71 (dd, *J* = 8.5, 2.0 Hz, 1H), 7.45–7.41 (m, 2H), 7.32 (s, 1H), 7.20 (t, *J* = 7.4 Hz, 1H), 6.96 (d, *J* = 8.4 Hz, 1H), 4.06 (s, 3H), 3.99 (s, 3H), 2.35 (s, 3H).

Calculated for: C_19_H_18_N_2_O_3_ C 70.79; H 5.63; N 8.69 Found 70.63; H 5.46; N 8.48.

#### 3.2.2. A Synthesis of Selected 4,4′-(4-Arylmethylene)-bis-(3-methyl-1-phenylpyrazol-5-oles) from Aldehydes 2 and 5-Methyl-2-phenyl-2,4-dihydro-3*H*-pyrazole-3-one **6b**—General Procedure

4,4′-(4-Methylphenylmethylene)-bis-(3-methyl-1-phenylpyrazol-5-ol) **14a**.

A round-bottomed flask (50 mL) equipped with a reflux condenser and a magnetic stirring bar was charged with *p*-tolu-aldehyde (120 mg, 1 mmol), 5-methyl-2-phenyl-2,4-dihydro-3*H*-pyrazole-3-one (340 mg, 2 mmol) and ethanol (10 mL). The content was boiled for five hours. After a few minutes, a formation of light pink precipitate was observed. After cooling, the precipitate was filtered off and crystallised from the ethanol/DMF mixture.

Pale pink crystals, 350 mg, 77%, mp. 204–205 °C. The ^1^H NMR/^13^C NMR spectra are included in Section 3.2.1.

4,4′-(4-Hydroxyphenylmethylene)-bis-(3-methyl-1-phenylpyrazol-5-ol) **14h**.

After cooling the solution was evaporated and the resulted solid was crystallised from methanol. Light yellow crystals, 375 mg, 82%, mp. 158–160 °C. Lit. 153–155 °C [30]. The ^1^H NMR/^13^C NMR spectra are included in Section 3.2.1.

4,4′-(3,4-Dimethoxyphenylmethylene)-bis-(3-methyl-1-phenylpyrazol-5-ol) **14i**

Pale yellow crystals, 425 mg, 85%, mp. 208–209 °C. The ^1^H NMR/^13^C NMR spectra are included in Section 3.2.1.

4,4′-(2-Furylmethylene)-bis-(3-methyl-1-phenylpyrazol-5-ol) **14j**.

Colourless crystals, 278 mg, 65%, mp. 187–189 °C. Lit. 188–189 °C [27].

^1^H NMR (400 MHz, DMSO-d_6_,) δ, ppm (*J*, Hz): 13.85 (s, 1H), 7.72 (d, *J* = 7.7 Hz, 4H), 7.51 (s, 1H), 7.45 (t, *J* = 7.9 Hz, 4H), 7.26 (t, *J* = 7.4 Hz, 2H), 6.35 (dd, *J* = 3.1, 1.9 Hz, 1H), 6.13 (d, *J* = 3.2 Hz, 1H), 4.99 (s, 1H), 2.31 (s, 6H) [27].^13^C NMR (101 MHz, DMSO-d_6_) δ, ppm: 154.59, 146.42, 142.01, 129.38, 126.06, 121.02, 110.83, 106.60, 28.73, 11.96.

#### 3.2.3. Synthesis of 4-Aryl-4,9*H*-dihydro-3-methyl-1-phenyl-1*H*-pyrazolo[3,4-*b*]quinolines **13** by Cyclisation of 3-Methyl-*N*,1-diphenyl-1*H*-pyrazol-5-amine **19** with Aldehydes **2**

3-Oxo-*N*-phenylbutanethioamide **17**.

The compound was prepared according to the literature procedure [36].

3-Methyl-*N*,1-diphenyl-1*H*-pyrazol-5-amine **19**.

The compound was prepared by reacting **17** with phenyl-hydrazine **18** according to literature procedure [37].

A glass vial (10 mL) equipped with a magnetic stirring bar was charged with anhydrous zinc chloride (200 mg, 1.5 mmol), *p*-tolu-aldehyde **2a** (180 mg, 1.5 mmol) and **19** (380 mg, 1.5 mmol). The vial was closed with an air condenser and inserted into an aluminium heating block and heated at 125 °C for 6 h whilst stirring. After cooling, the melt was digested with ethanol, sonicated and filtered off to remove pyrazolo[3,4-*b*]quinoline **7a**. A filtrate was evaporated and the resulting oil was dissolved in toluene and chromatographed on a column packed with silica gel (Merck 60, 70–230 mesh) using a toluene–ethyl acetate mixture as an eluent (toluene: AcOEt/3:0.1→3:0.4) to separate **13a** from **21a**. The experiment was performed in a two-fold way: in an open vial and with a closed vial.

4-(4-Methylphenyl)-3-methyl-1-phenyl-1*H*-pyrazolo[3,4-*b*]quinoline **7a**.

Yellow crystals, 150 mg, 26.7% (an open vial), 35 mg, 6.3% (a closed vial), mp. 208–210 °C. Lit. 206–207 °C [11].

^1^H NMR (400 MHz, CDCl_3_) δ, ppm (*J*, Hz): 8.55 (d, *J* = 7.7 Hz, 2H), 8.23 (d, *J* = 8.1 Hz, 1H), 7.77 (t, *J* = 7.7 Hz, 4H), 7.59 (dd, *J* = 8.4, 7.6 Hz, 4H), 7.40 (dt, *J* = 13.7, 8.1 Hz, 5H), 7.31 (t, *J* = 7.4 Hz, 1H), 2.56 (s, 3H), 2.20 (s, 3H). ^13^C NMR (101 MHz, CDCl_3_) δ, ppm: δ 150.18, 148.49, 144.76, 143.97, 140.02, 138.62, 131.93, 130.25, 129.64, 129.03, 128.94, 127.06, 124.88, 123.81, 120.33, 116.46, 21.46, 15.07.

4-(4-Methylphenyl)-4,9-dihydro-3-methyl-1-phenyl-1*H*-pyrazolo[3,4-*b*]quinoline **13a**

Colourless crystals, 67 mg, 11.6% (open vial), 174 mg, 30.1% (closed vial), m.p. 178–180 °C.

^1^H NMR (400 MHz, CDCl_3_) δ, ppm (*J*, Hz): 7.60–7.54 (m, 2H); 7.54–7.47 (m, 2H); 7.34 (t, *J* = 7.3 MHz, 1H), 7.15 (d, *J* = 8.1 Hz, 2H); 6.86–6.79 (m, 1H); 6.74 (dd, *J* = 8.4; 0.9 Hz, 1H); 6.29 (s, 1H, N-H), 5.29 (s, 1H), 2.31 (s, 3H); 1.95 (s, 3H). ^13^C NMR (100 MHz, CDCl_3_) δ, ppm: 147.72; 144.35; 138.58; 138.55, 137.20; 135.95; 131.32; 129.90, 129.33; 128.23; 127.25; 126.98; 125.00; 122.79; 122.13; 115.90; 100.77; 42.06, 21.15, 12.66.

Calculated for C_24_H_21_N_3_ ESI (M+H^+^) = 352,1808. Measured: HRMS/ESI (M+H^+^) = 352,1810.

4,4′-(4-Metylphenylmethylene)-bis-[3-methyl-*N*,1-diphenyl-1*H*-pyrazol-5-amine] **21a**.

Colourless crystals, 218 mg (closed vial), 45%, mp. 198–199 °C.

^1^H NMR( 400 MHz, CDCl_3_) δ, ppm (*J*, Hz): 7.45 (d, *J* = 7.9 Hz, 4H); 7.30–7.21 (m, 5H); 7.16 (t, *J* = 7.4 Hz, 2H); 7.03–6.82 (m, 7H); 6.66 (t, *J* = 7.3 Hz, 2H); 6.26 (d, *J* = 7.8 Hz, 4H); 5.18 (s, 1H); 4.58 (s, 2H), 2.24 (s, 3H); 1.97 (s, 6H). ^13^C NMR (101 MHz, CDCl_3_) δ, ppm: 148.33; 144.71; 139.07; 137.37; 136.09; 129.09; 128.89; 128.18; 126.68; 122.92; 119.62; 115.07; 113.91; 36.34 (C**H-**methylene); 21.06 (C_6_H_4_C**H_3_**); 13.94 (3-CH_3_).

Cald. for C_40_H_36_N_6_: **ESI** (M+H^+^) = 601,3074. Measured: HRMS/ESI (M+H^+^) = 601,3072.

4-(*p*-Chlorophenyl)-1-phenyl-3-methyl-1*H*-pyrazolo[3,4-*b*]quinoline **7b**.

Yellow crystals, 32 mg, 5.4%, mp. 230–231 °C. The sample was identified with the original one prepared according to the literature procedure [38].

^1^H NMR (400 MHz, CDCl_3_) δ, ppm (*J*, Hz): 8.41 (dt, *J* = 8.7, 1.6 Hz, 2H); 8.14–8.09 (m, 1H); 7.67 (ddd, *J* = 10.1, 5.8, 2.5 Hz, 1H); 7.59 (dt, 8.6, 4.3 Hz, 1H); 7.53–7.45 (m, 4H); 7.36–7.32 (m, 2H); 7.30 (ddd, *J* = 8.5, 6.6, 1.2 Hz, 1H), 7.23–7.18 (m, 1H); 2.10 (s, 3H). ^13^C NMR (100 MHz, CDCl_3_) δ, ppm:150.07; 148.30; 143.45; 142.86; 139.87; 135.07; 133.46; 131.11; 130.44; 129.10; 128.66; 126.58; 125.08; 124.21; 123.42; 120.39; 116.23; 15.15.

4-(*p*-Chlorophenyl)-4,9-dihydro-1-phenyl-3-methyl-1*H*-pyrazolo[3,4-*b*]quinoline **13b**

Pale yellow powder, 53 mg, 8.9%, m.p. 129–130 °C.

^1^H NMR (400 MHz, CDCl_3_) δ, ppm (*J*, Hz): 7.62–7.57 (m, 2H); 7.57–7.49 (m, 2H); 7.41–7.34 (m, 1H); 7.32–7.26 (m, 1H); 7.26–7.20 (m, 1H); 7.15–7.07 (m, 1H); 7.03(t, *J* = 7.5 Hz, 1H); 6.91–6.83 (m, 1H); 6.78 (dd, *J* = 8.0 Hz; 0.8 Hz, 1H), 6.41 (s, 1H, N-H); 5.34 (s, 1H, C-H); 1.98 (s, 3H, 3-CH_3_). ^13^C NMR (100 MHz, CDCl_3_) δ, ppm:147.42; 145.72; 138.52; 137.10; 132.16; 131.14; 129.82; 129.52; 128.48; 127.40; 126.98; 124.15; 122.69; 122.08, 115.89; 100.05; 41.11; 12.59.

Calcd for C_23_H_18_ClN_3_ C 74.29; H 4.88; N 11.30. Found C 74.03; H 4.56; N 11.18.

4,4′-(4-Chlorophenylmethylene)-bis-[3-methyl-*N*,1-diphenyl-1*H*-pyrazol-5-amine)] **21b**.

White powder, 222 mg, 44.5%, mp. 202–203 °C.

^1^H NMR (400 MHz, CDCl_3_) δ, ppm: 7.51–7.43 (m, 4H); 7.31 (t, *J* = 7.8 Hz, 4H); 7.21(t, *J* = 7.4 Hz, 2H); 7.10 (d, *J* = 8.5 Hz, 2H); 7.02 (dd, *J* = 16.5 Hz, 8.7 Hz, 6H); 6.72 (t, *J* = 7.4 Hz, 2H); 6.29 (d, *J* = 7.7 Hz, 4H); 5.20 (s, 1H); 4.59 (s, 2H); 2.02 (s, 6H). ^13^C NMR (100 MHz, CDCl_3_) δ, ppm: 148.00; 144.58; 139.04; 138.83; 137.26; 132.21; 129.37; 129.07; 129.01; 128.55; 126.78; 122.92; 119.69; 114.66; 113.74; 36.21; 13.91.

Calcd for C_39_H_33_ClN_6_ C 75.41; H 5.35; N 13.53 Found C 75.32; H 5.23; N 13.48.

4-(2,4-Dichlorophenyl)-1-phenyl-3-methyl-1*H*-pyrazolo[3,4-*b*]quinoline **7e**

In this procedure 300 mg of **20** and 210 mg of 2,4-dichlorobenzaldehyde were used for reaction.

Yellow crystals, 24 mg (closed vial), 4.9%, m.p. 158–159 °C.

^1^H NMR (400 MHz, CDCl_3_) δ, ppm (*J*, Hz): 8.54 (dd, *J* = 8.7; 1.1 Hz, 2H); 8.26 (d, *J* = 8.3 Hz, 1H); 7.83–7.77 (m, 1H); 7.72 (d, *J* = 2.0 Hz, 1H); 7.62–7.50 (m, 2H); 7.46–7.40 (m, 1H), 7.38 (d, *J* = 8.2 Hz, 1H); 7.35–7.29 (m, 1H); 2.22 (s, 3H, 3-Me). ^13^C NMR (100 MHz, CDCl_3_) δ, ppm: 150.13; 148.53; 143.17; 139.85; 139.55; 135.85; 134.73; 132.69; 132.14; 130.45; 129.73; 129.28; 129.06; 127.24; 126.00; 125.09; 124.55; 122.92; 120.33; 116.31; 14.17.

Calcd for C_23_H_15_Cl_2_N_3_ C 68.33; H 3.74; N 10.39. Found C 68.14; H 3.45; N 10.28.

4-[*p*-(2,4-Dichlorophenyl)]-4,9-dihydro-1-phenyl-3-methyl-1*H*-pyrazolo[3,4-*b*]quinoline **13e**

Pale yellow crystals, 128 mg, 27%, m.p. 220–221 °C.

^1^H NMR (400 MHz, CDCl_3_) δ, ppm: 7.62–7.57 (m, 2H); 7.54 (dd, *J* = 10.5 Hz, 5.2 Hz, 2H); 7.44 (t, *J* = 1.1 Hz, 1H); 7.41–7.35 (m, 1H); 7.16 (d, *J* = 1.1 Hz, 2H); 7.14–7.09 (m, 1H); 7.08 (d, *J* = 7.7 Hz, 1H); 6.90–6.84 (m, 1H); 6.78 (dd, *J* = 8.0 Hz, 0.8 Hz, 1H); 6.42 (s, 1H, N-H); 5.99 (s, 1H, C-H); 1.97 (s, 3H). ^13^C NMR (100 MHz, CDCl_3_) δ, ppm: 147.37; 143.43; 138.76; 138.42; 137.10; 132.76; 132.52; 132.43; 130.74; 129.83; 128.88; 128.11; 127.66; 127.03; 123.65; 122.48; 122.24; 115.90; 99.69; 36.85; 11.91.

Calcd for C_23_H_17_Cl_2_N_3_ C 67.99; H 4.22; N 10.34. C 67.81; H 4.08; N 10.17.

4-(Thienyl-2-ylo)-3-methyl-1-phenyl-1*H*-pyrazolo[3,4-*b*]quinoline **7d**

Yellow crystals, 95 mg (closed vial), 17%, m.p. 141–142 °C.

^1^H NMR (400 MHz, CDCl_3_) δ, ppm (*J*, Hz): 8.48 (dd, *J* = 8.7 Hz, 1.1 Hz, 2H); 8.17 (d, *J* = 7.8 Hz, 1H); 7.89 (d, *J* = 8.6 Hz, 1H); 7.77–7.71 (m, 1H); 7.64 (dd, *J* = 5.1 Hz, 1.2 Hz, 1H); 7.58–7.51 (m, 2H); 7.43–7.38 (m, 1H); 7.31–7.23 (m, 3H), 2.3 (s, 3H). ^13^C NMR (101 MHz, CDCl_3_) δ, ppm: 153.47; 146.80; 139.27; 139.17; 138.29; 130.89; 129.93; 127.76; 126.99; 126.82; 125.33; 124.10; 123.65; 123.06; 121.50; 117.28; 99.95; 36.85; 1267.

Calcd for: C_21_H_15_N_3_S C 73.87; H 4.43; N 12.31. Found C 73.57; H 4.28; N 12.18.

4-(Thienyl-2-ylo)-4,9-dihydro-3-methyl-1-phenyl-1*H*-pirazolo[3,4-*b*]quinoline **13g**

Colourless crystals, 150 mg, 29%, m.p. 274–276 °C.

^1^H NMR (400 MHz, CDCl_3_) δ, ppm: 9.02 (s, 1H); 7.58–7.46 (m, 4H); 7.32 (t, *J* = 7.2 Hz, 1H); 7.26 (t, *J* = 7.5 Hz, 1H); 7.12 (d, *J* = 7.6 Hz, 1H); 7.04 (d, *J* = 4.1 Hz, 2H); 6.98 (d, *J* = 4.1Hz, 1H); 6.88 (dd, *J* = 5.0 Hz, 3.5 Hz, 1H); 6.83–6.75 (m, 1H); 5.67 (s, 1H); 1.90 (s, 3H). ^13^C NMR (100 MHz, CDCl_3_) δ, ppm: 153.47; 146.80; 139.27; 139.17; 138.29; 130.89; 129.93; 127.76; 126.99; 126.82; 125.42; 124.10; 123.65; 123.06; 121.50; 117.28; 99.95; 36.85; 12.67.

Calculated for C_21_H_15_N_3_S: ESI (M+H^+^) = 344,1216. Measured: HRMS/ESI (M+H^+^) = 344,1212.

## 4. Conclusions

The aim of our research was to investigate the possibility of obtaining fully aromatic 4-aryl-1*H*-pyrazolo[3,4-*b*]quinolines by the oxidation of 4-aryl-4,9-dihydro-1*H*-pyrazolo[3,4-*b*]quinolines. These last compounds, according to the paper published by Hegde and Shetty that we investigated some time ago, can be easily obtained in a three-component reaction from aromatic aldehyde, aniline, pyrazolone and a catalytic amount of L-proline. Unfortunately, after repeating the procedures described in the above-mentioned publication, we failed to obtain the expected compounds. The only compounds that we obtained were 4,4′-(arylenemethylene)-bis-(3-methyl-1-phenyl-pyrazol-5-ol)-oles, regardless of the modification of the reaction conditions. Their structure was confirmed beyond any doubt by ^1^H NMR/^13^C NMR and literature studies. The actual 4-aryl-4,9-dihydro-1*H*-pyrazolo[3,4-*b*]quinolines were obtained by another synthetic method and used as standards in the original multi-component reaction. During tests, it turned out that they are not produced even in trace amounts. Therefore, we certainly disagree with the authors’ final statement: “In conclusion, a simple and efficient protocol for one-pot multi-component synthesis of 1H-pyrazolo[3,4-*b*]quinolines using L-proline as a catalyst has been developed” [18]. It seems to us that the authors simply misinterpreted the data they obtained.

## Figures and Tables

**Table 1:** ^1^H NMR data of “4-aryl-4,9-dihydro-1*H*-pyrazolo[3,4-*b*]quinolines” **13** and 4,4′-(arylene-methylene)-bis-(3-methyl-1-phenyl-pyrazol-5-oles) **14**.

Ar	“4-Aryl-4,9-dihydro-1*H*-pyrazolo[3,4-*b*]quinoline” 13 [18]		4,4′-(Arylenemethylene)-bis-(3-methyl-1-phenyl-pyrazol-5-ol) 14	
	N-H	C-H	O-H	C-H
MeC_6_H_4_-	14.00	4.89	13.94; 12.44	4.92 [26]
ClC_6_H_4_-	13.90	4.96	13.89; 12.53	4.98 [26]
FC_6_H_4_-	13.92	4.77	13.70	4.94 [27]
MeOC_6_H_4_-	13.92	4.96	13.89	5.09 [27]
2,4-ClC_6_H_3_-	13.93	5.09	13.95; 12.67	5.05 [28]
NO_2_C_6_H_4_-	13.03	4.96	13.90; 12.49	5.14 [28]
3,4-OMeC_6_H_3_-	13.92	4.89	14.11; 11.68	4.91 [29]
4-OHC_6_H_4_	13.92	4.98	13.96; 12.2	4.86 [30]
Thiophen-2-yl	13.91	4.96	14.01; 12.51	5.14 [26]
Furan-2-yl	13.89	4.96	13.24	5.01 [27]

## Data Availability

All data obtained and analysed during this study are included in the published article.

## Supplementary Materials

The following supporting information can be downloaded at: https://www.mdpi.com/article/10.3390/molecules28227612/s1, The ^1^H NMR and ^13^C NMR data of investigated compounds are available online.
