# Supplementary material for: Multicomponent Synthesis of 4-Aryl-4,9-dihydro-1H-pyrazolo[3,4-b]quinolines Using L-Proline as a Catalyst—Does It Really Proceed?
Source: Molecules. 2023 Nov 15;28(22):7612. doi: 10.3390/molecules28227612 (PMC10673494; doi:10.3390/molecules28227612)
Supplement: Supplementary file 1 [file molecules-28-07612-s001.zip › molecules-2668177-supplementary.pdf]

Supplemantary materials

^{1}H NMR/^{13}C NMR spectra

Multicomponent synthesis of 4-aryl-4,9-dihydro-1*H*-pyrazolo[3,4-*b*]quinolines using L-proline as a catalyst – does it really proceed?

Andrzej Danel[1], Elżbieta Porębska[1], Kacper Markiel[1], Oleksii Havrysh[1], Mateusz Kucharek[2], Arkadiusz Gut[3], Tomasz Uchacz[3]

[1]Faculty of Materials Engineering and Physics, Krakow University of Technology, Podchorążych St.1, 30-084, Krakow, Poland; rrdanela@cyf-kr.edu.pl

[2]Faculty of Food Technology, Agricultural University, Balicka St. 122, 30-149 ,Krakow, Poland

[3]Faculty of Chemistry, Jagiellonian University, Gronostajowa St. 2, 30-387 ,Krakow, Poland

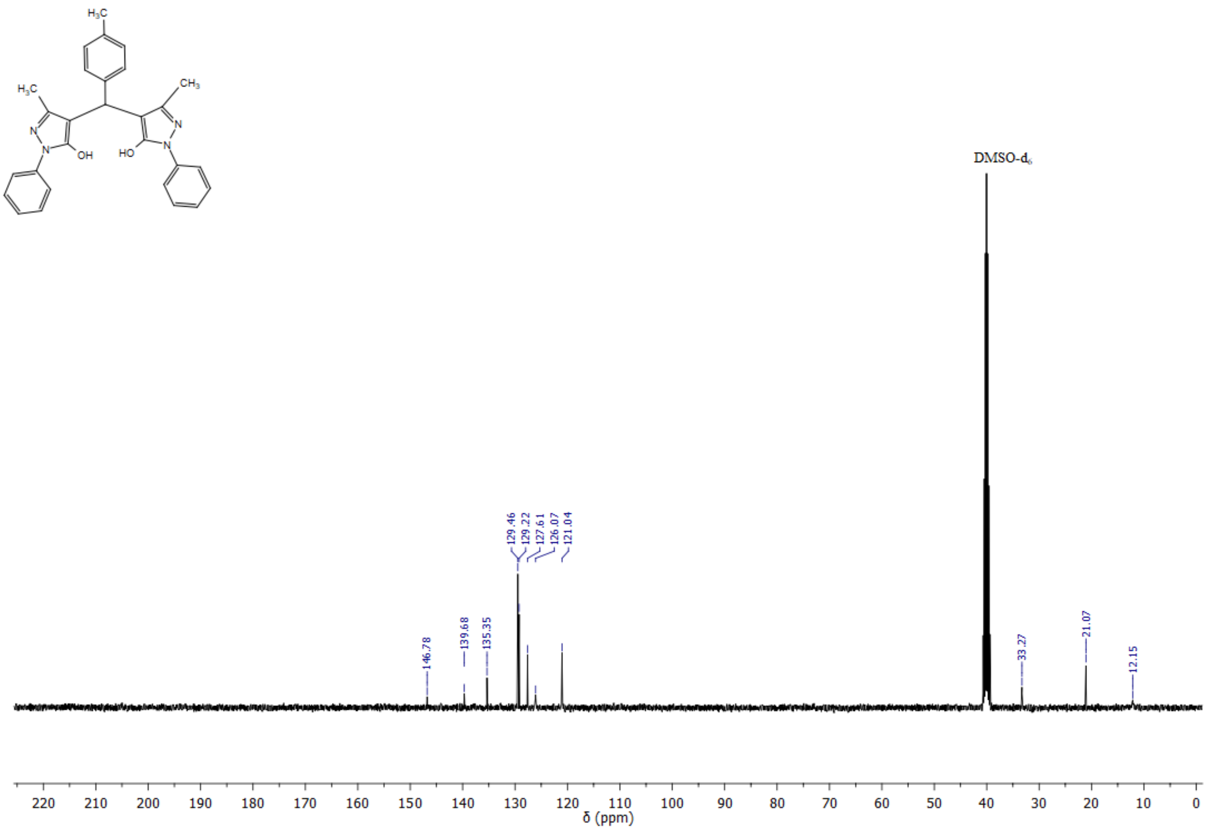

^{13}C NMR spectra of 4,4’-(4-Methylphenylmethylene)-bis-(3-methyl-1-phenylpyrazol-5-ol) **14a**

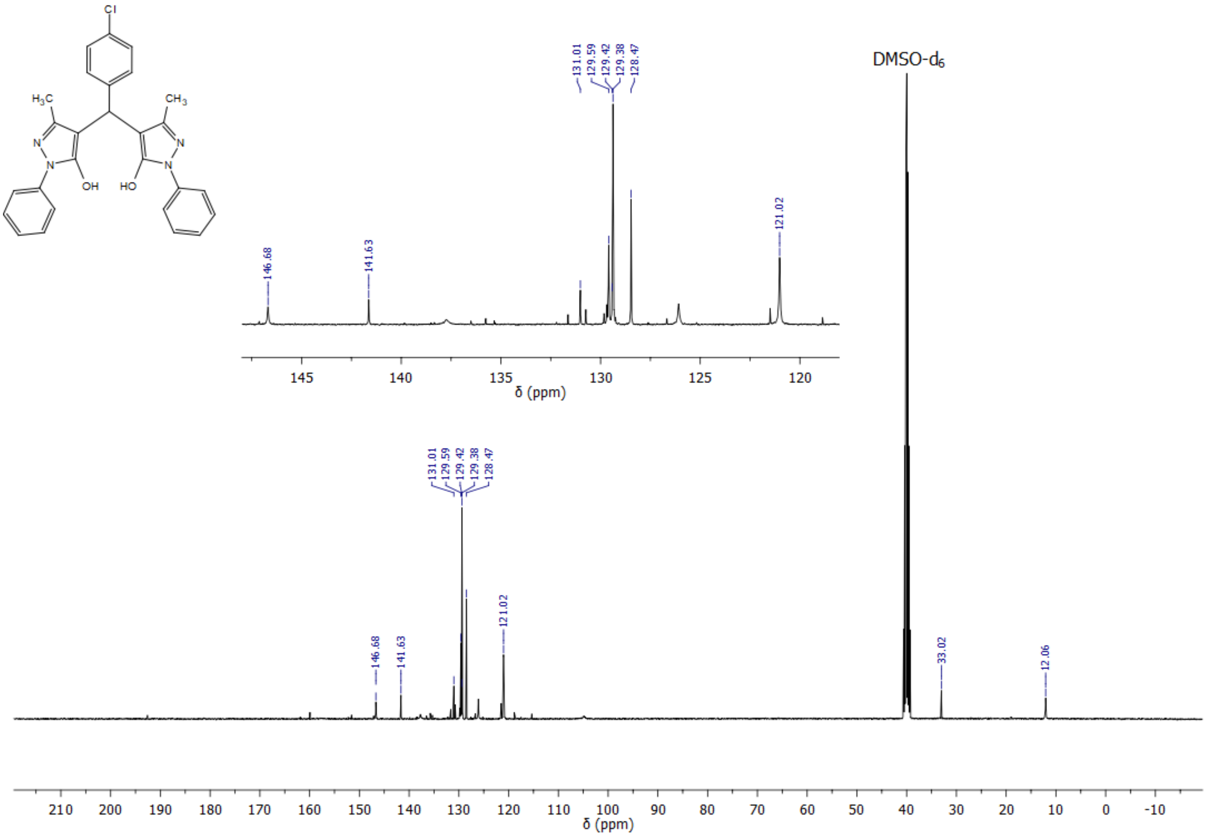

^{13}C NMR spectra of 4,4’-(4-Chlorophenylmethylene)-bis-(3-methyl-1-phenylpyrazol-5-ol) **14b**

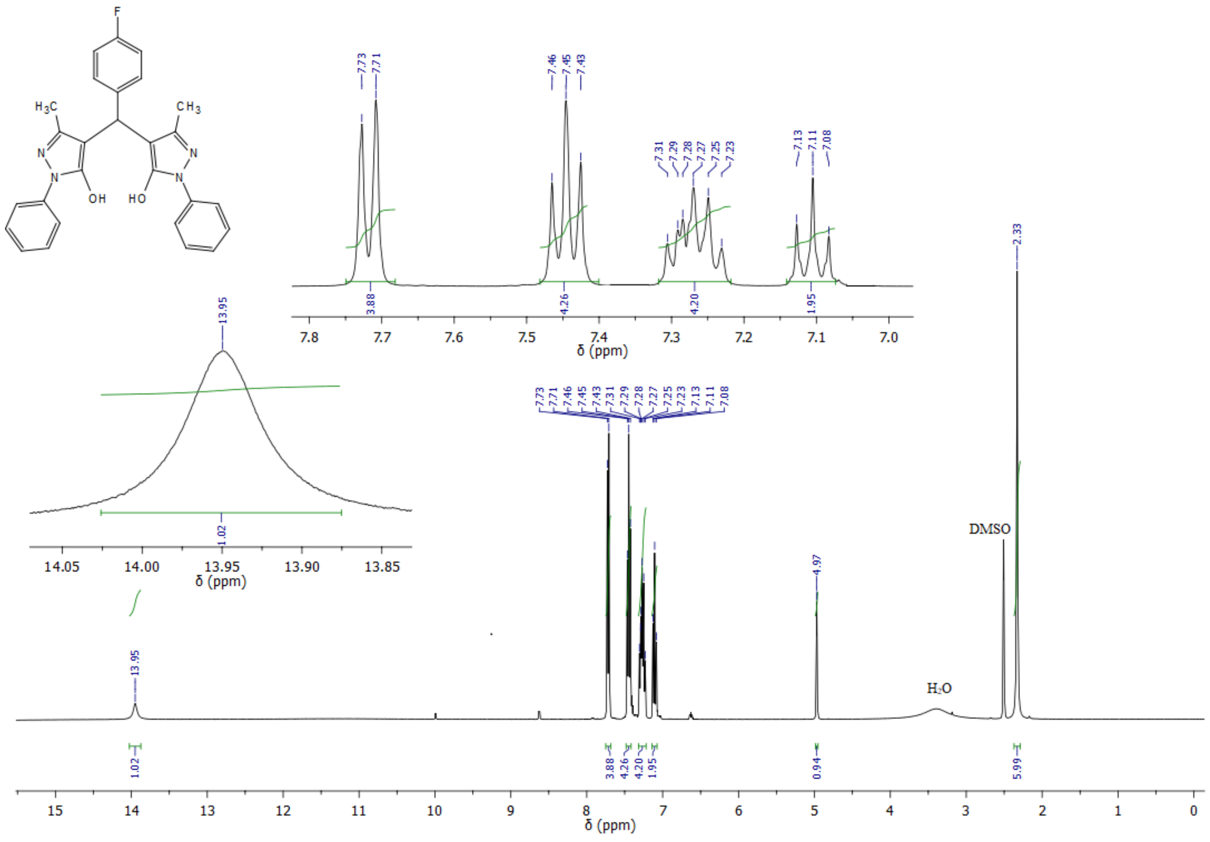

^{1}H NMR spectra of 4,4’-(4-Fluorophenylmethylene)-bis-(3-methyl-1-phenylpyrazol-5-ol) **14c**

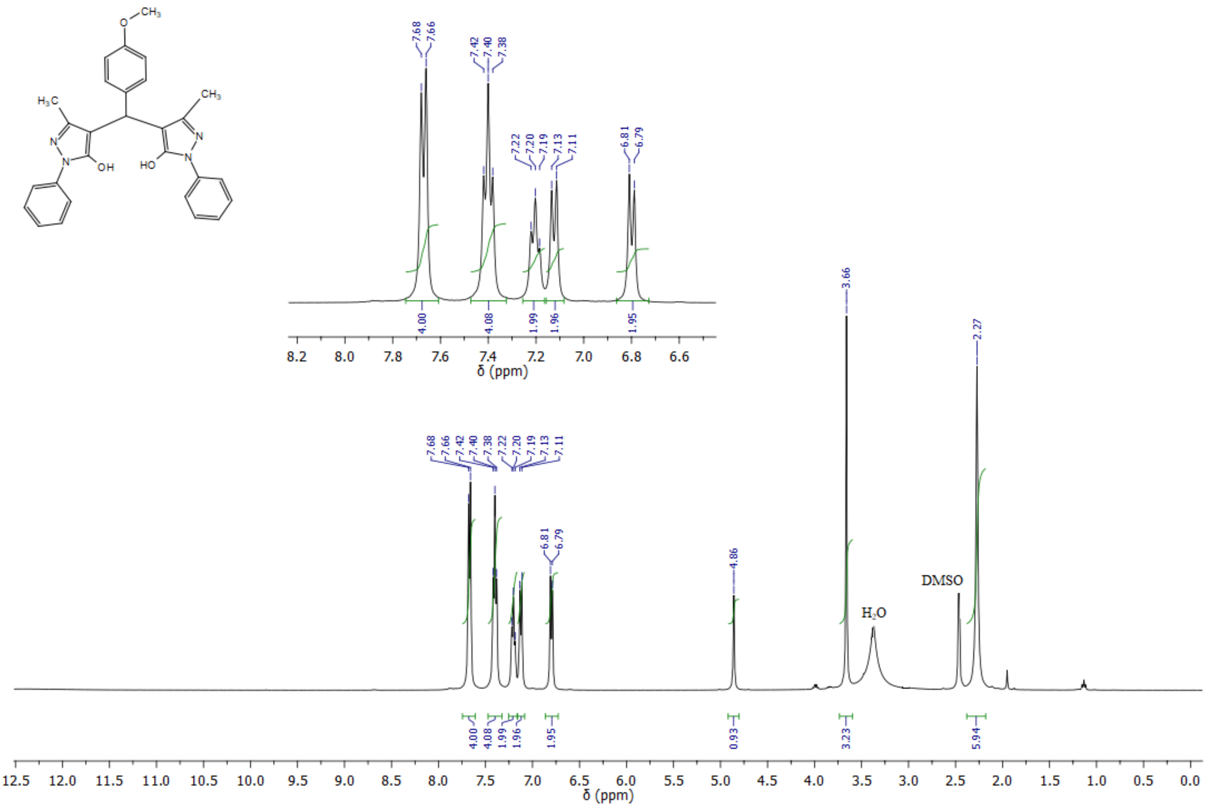

^{1}H NMR spectra of 4,4'-(4-Methoxyphenylmethylene)-bis-(3-methyl-1-phenylpyrazol-5-ol) **14d**

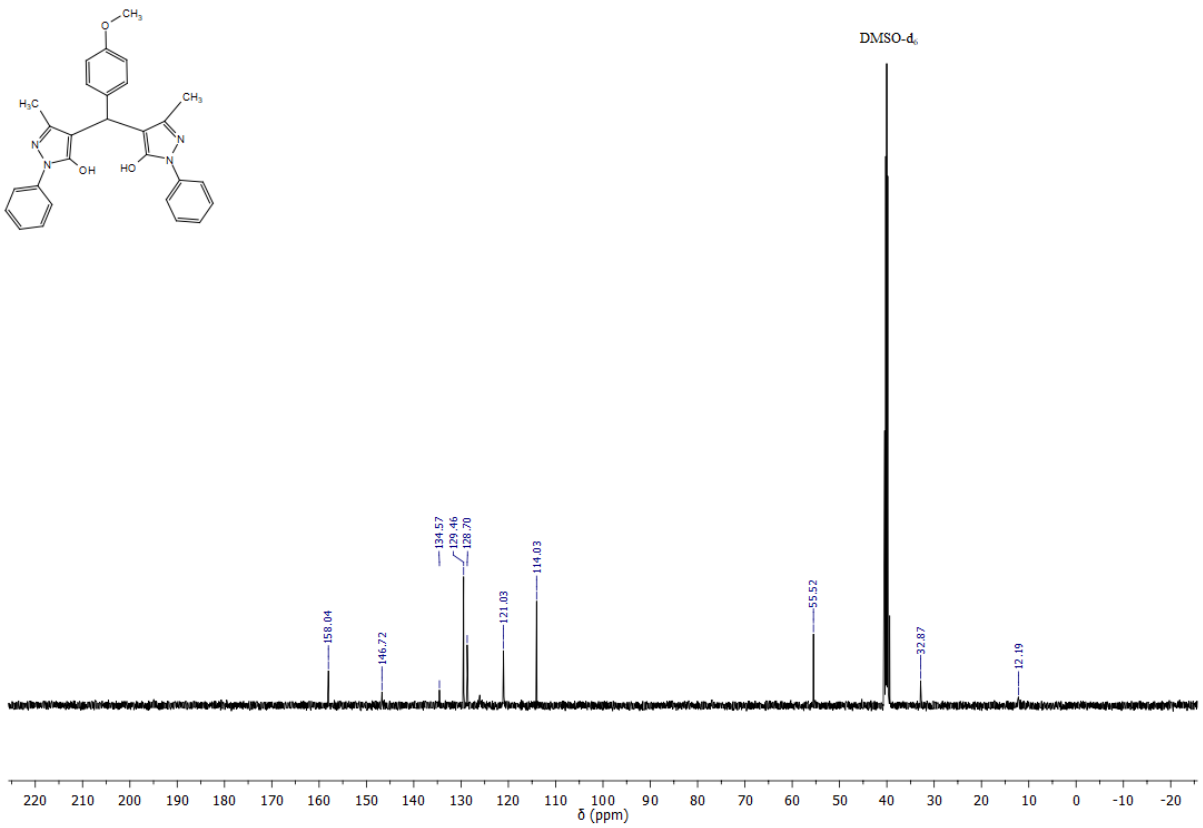

^{13}C NMR spectra of 4,4’-(4-Methoxyphenylmethylene)-bis-(3-methyl-1-phenylpyrazol-5-ol) **14d**

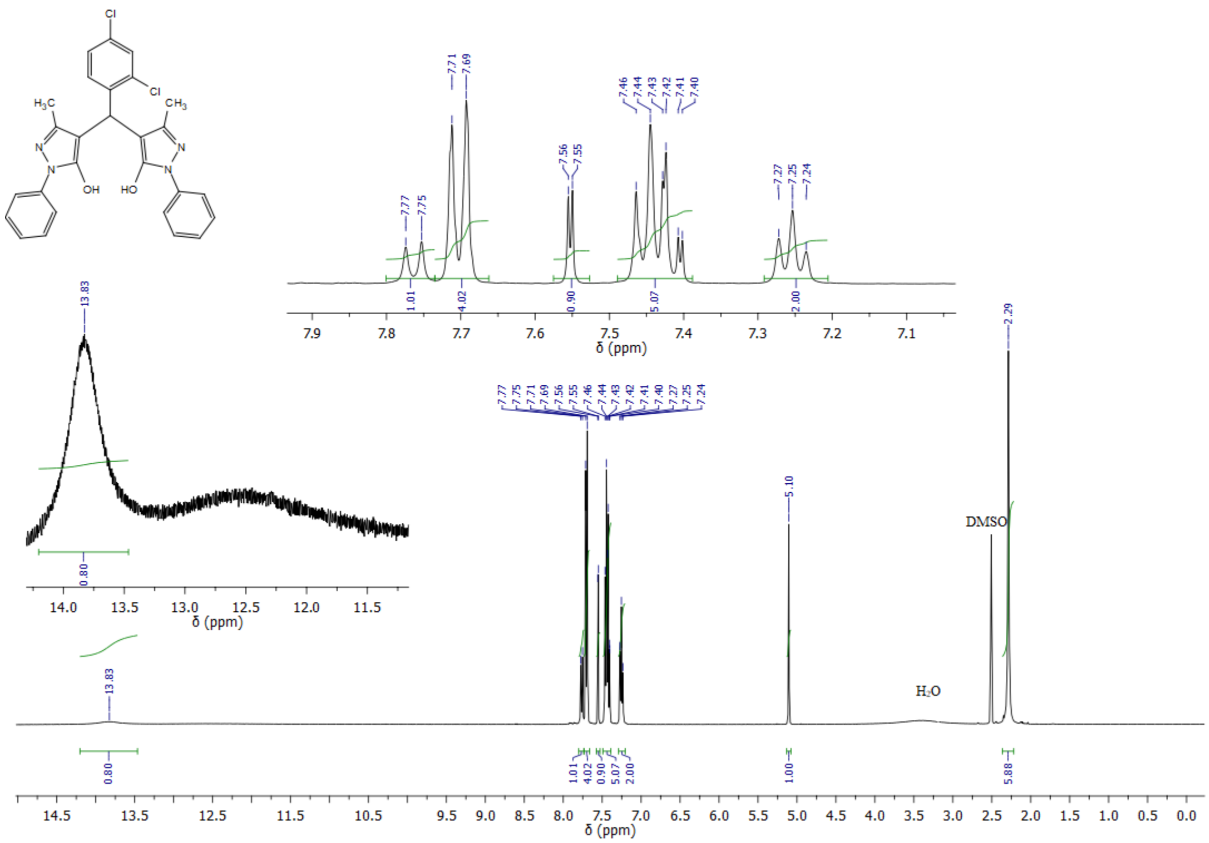

^{1}H NMR spectra of 4,4’-(2,4-Dichlorophenylmethylene)-bis-(3-methyl-1-phenylpyrazol-5-ol) **14e**

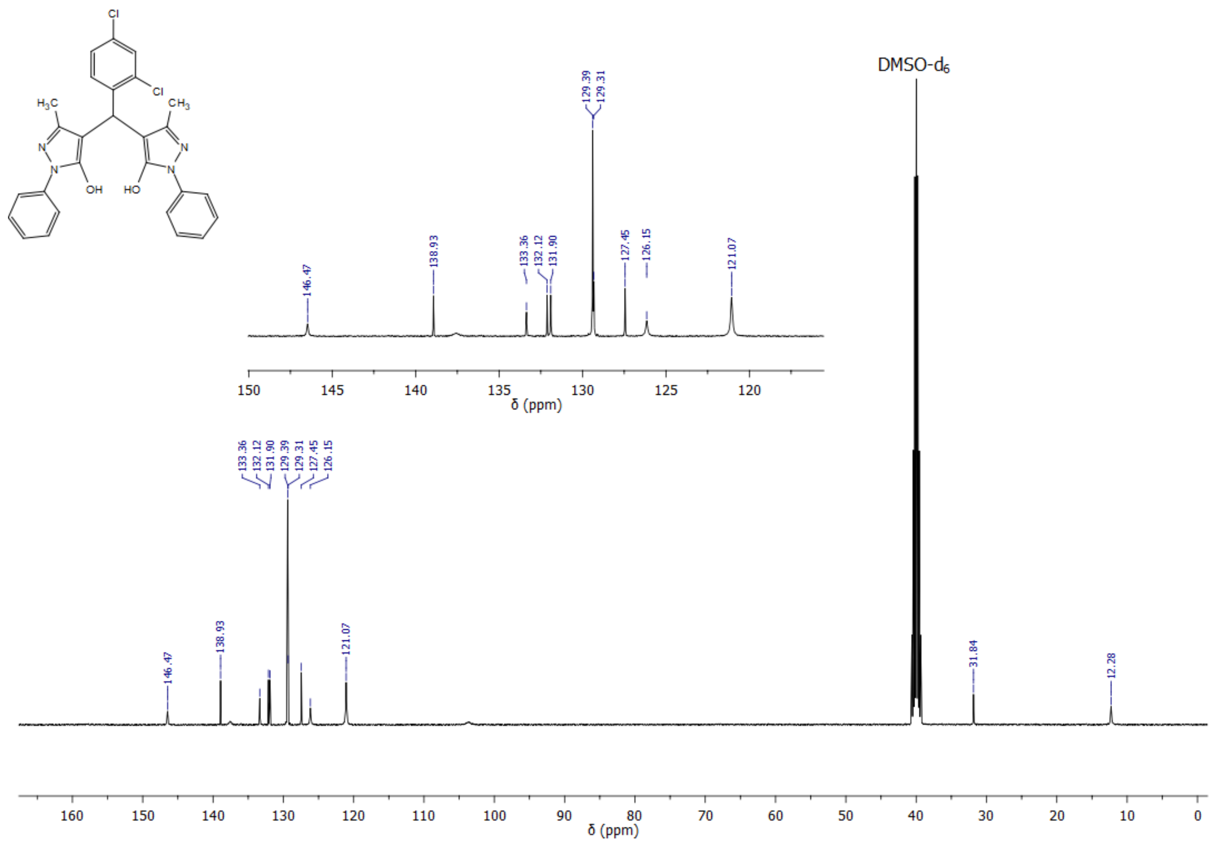

^{13}C NMR spectra of 4,4’-(2,4-Dichlorophenylmethylene)-bis-(3-methyl-1-phenylpyrazol-5-ol) **14e**

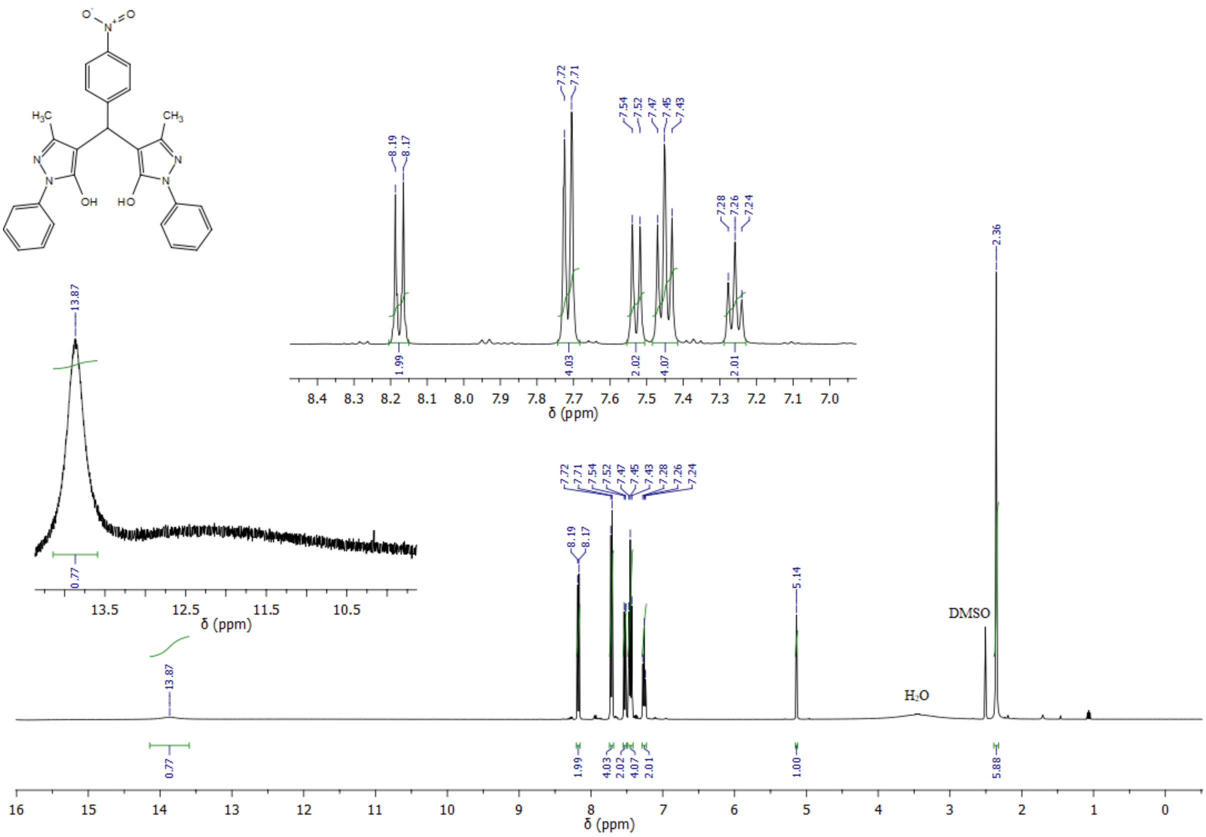

^{1}H NMR spectra of 4,4'-(4-Nitrophenylmethylene)-bis-(3-methyl-1-phenylpyrazol-5-ol) **14f**

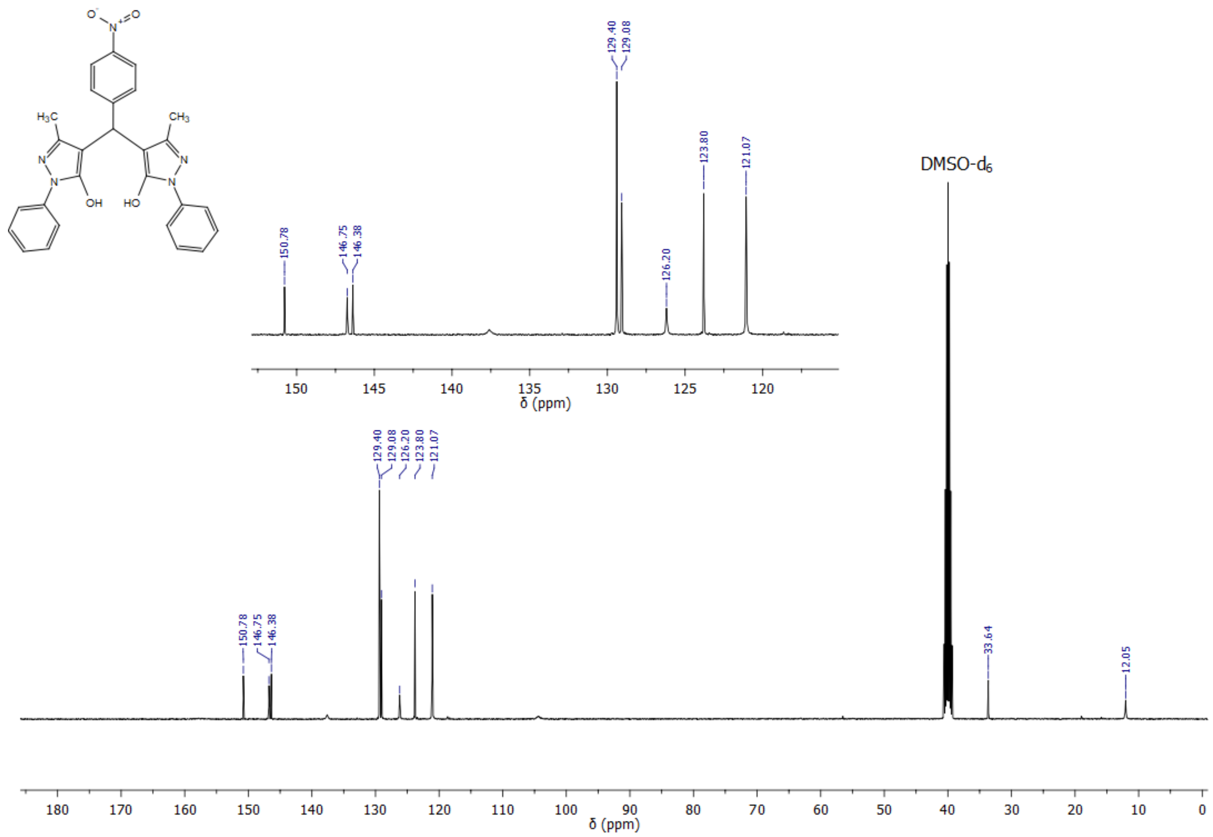

^{13}C NMR spectra of 4,4’-(4-Nitrophenylmethylene)-bis-(3-methyl-1-phenylpyrazol-5-ol) **14f**

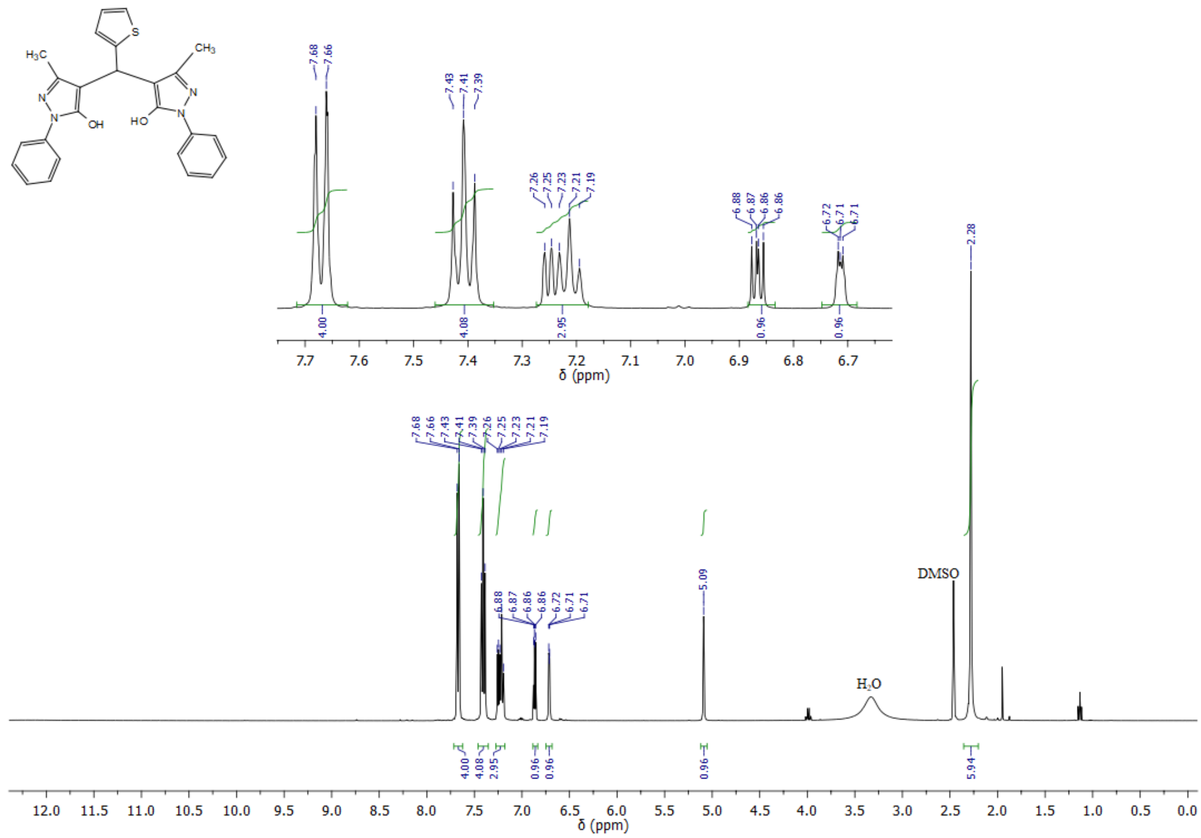

^{1}H NMR spectra of 4,4’-(2-Thienylmethylene)-bis-(3-methyl-1-phenylpyrazol-5-ol) **14g**

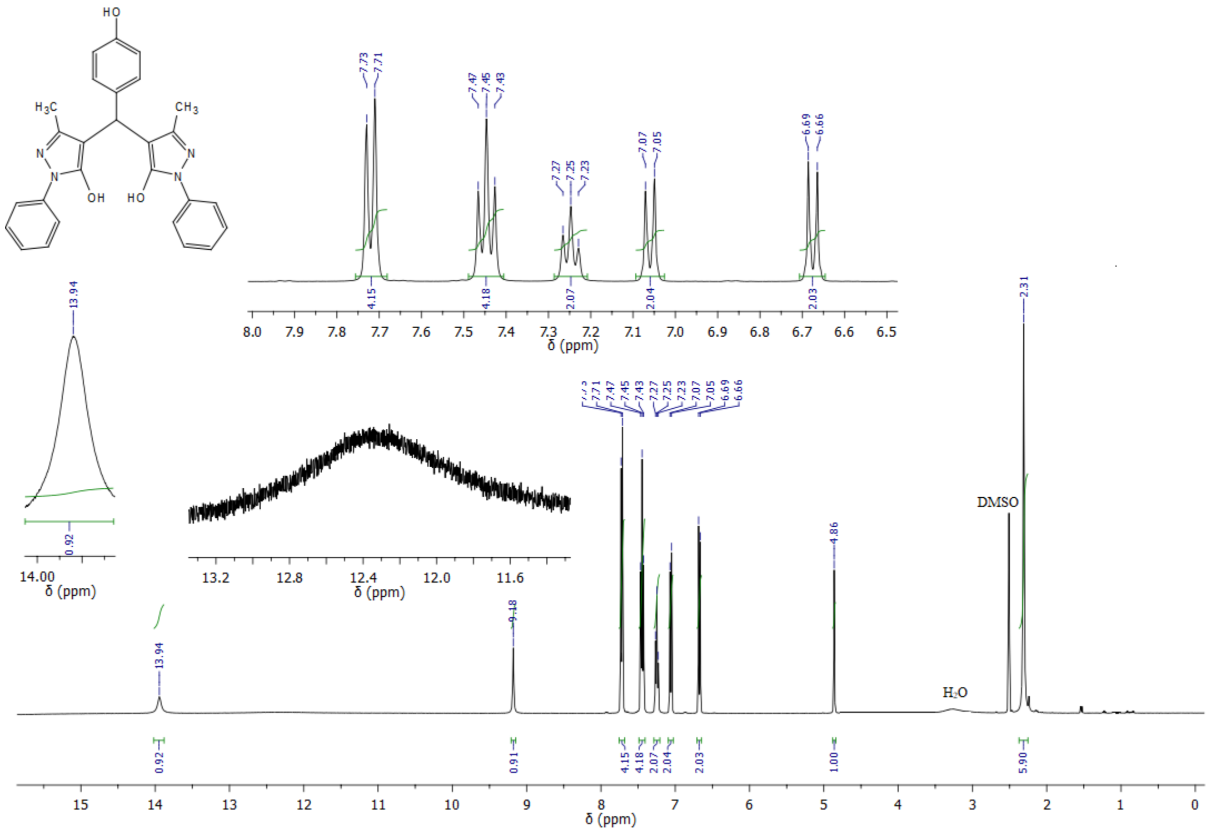

^{1}H NMR spectra of 4,4’-(4-Hydroxyphenylmethylene)-bis-(3-methyl-1-phenylpyrazol-5-ol) **14h**

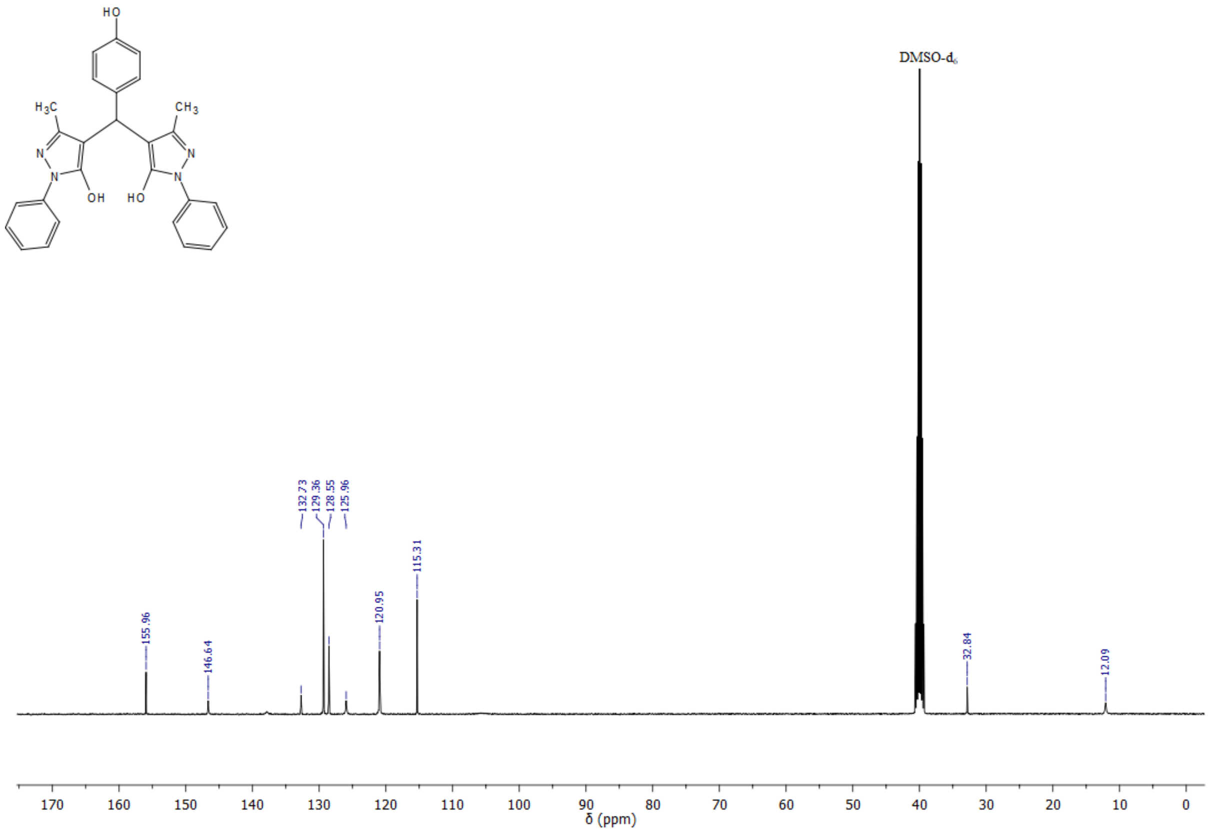

^{13}C NMR spectra of 4,4’-(4-Hydroxyphenylmethylene)-bis-(3-methyl-1-phenylpyrazol-5-ol) **14h**

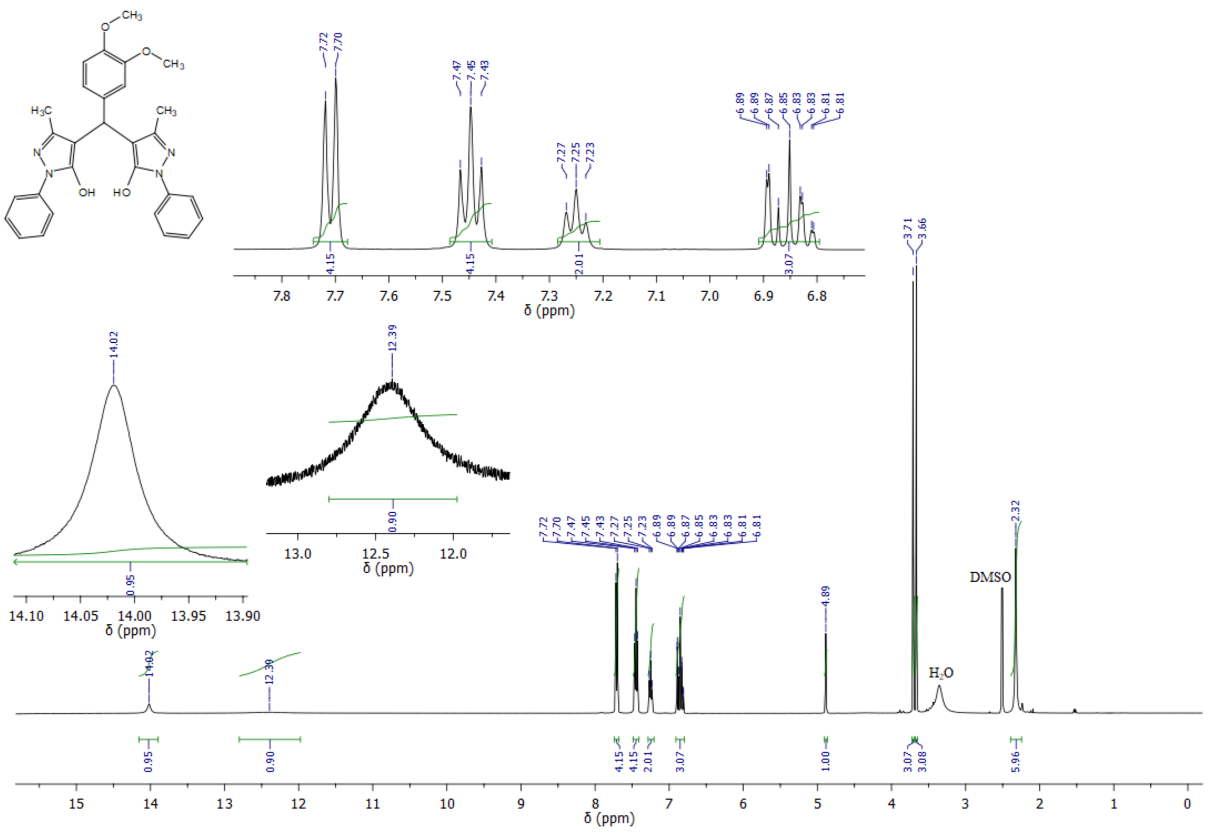

^{1}H NMR spectra of 4,4'-(3,4-Dimethoxyphenylmethylene)-bis-(3-methyl-1-phenylpyrazol-5-ol) **14i**

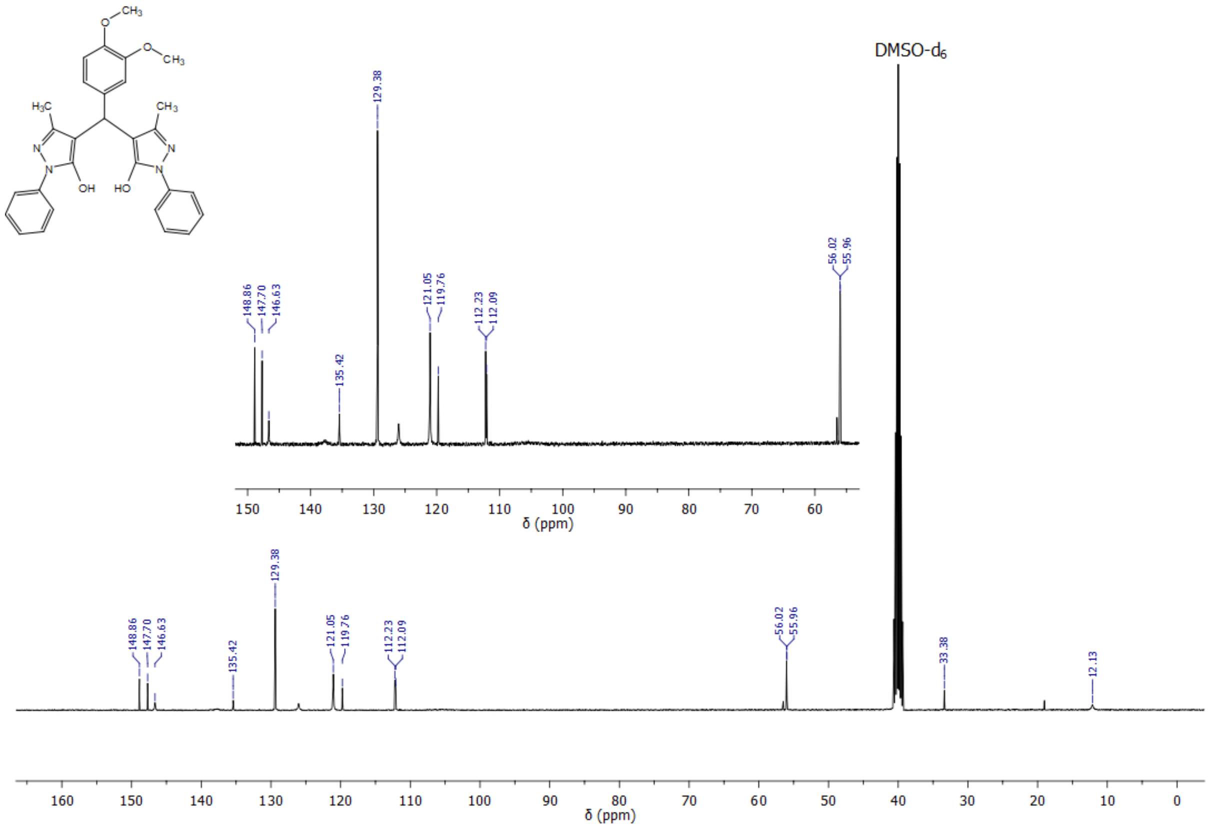

^{13}C NMR spectra of 4,4’-(3,4-Dimethoxyphenylmethylene)-bis-(3-methyl-1-phenylpyrazol-5-ol) **14i**

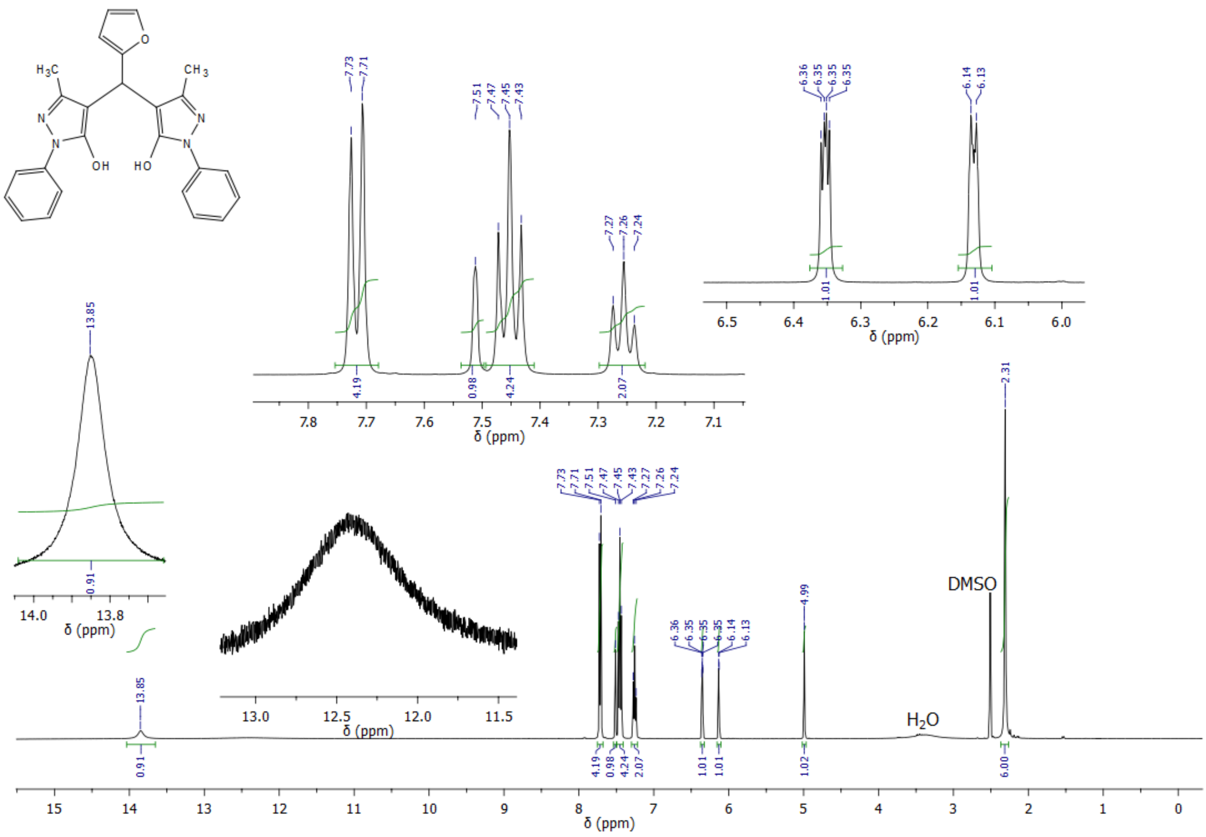

^{1}H NMR spectra of 4,4’-(Furan-2yl)methylene)-bis-(3-methyl-1-phenylpyrazol-5-ol) **14j**

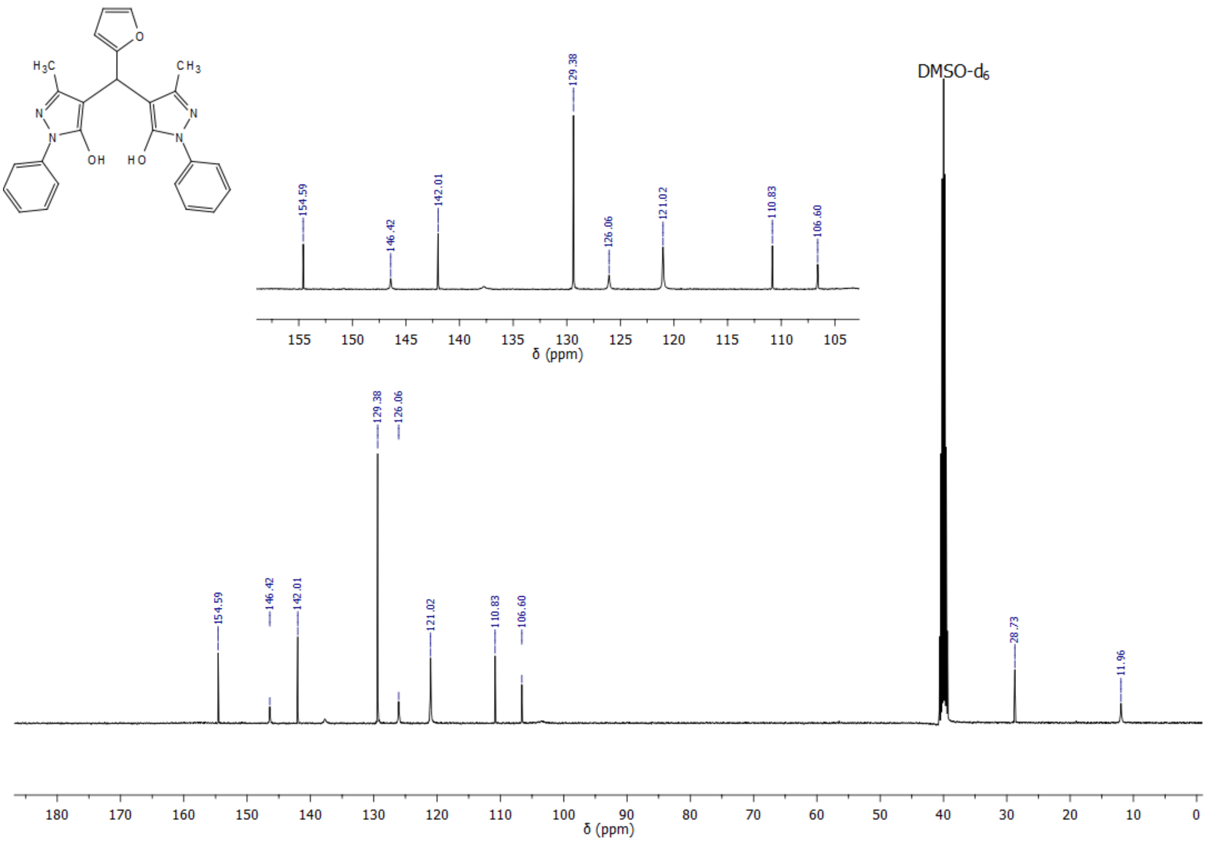

^{13}C NMR spectra of 4,4’-(Furan-2yl)methylene)-bis-(3-methyl-1-phenylpyrazol-5-ol) **14j**

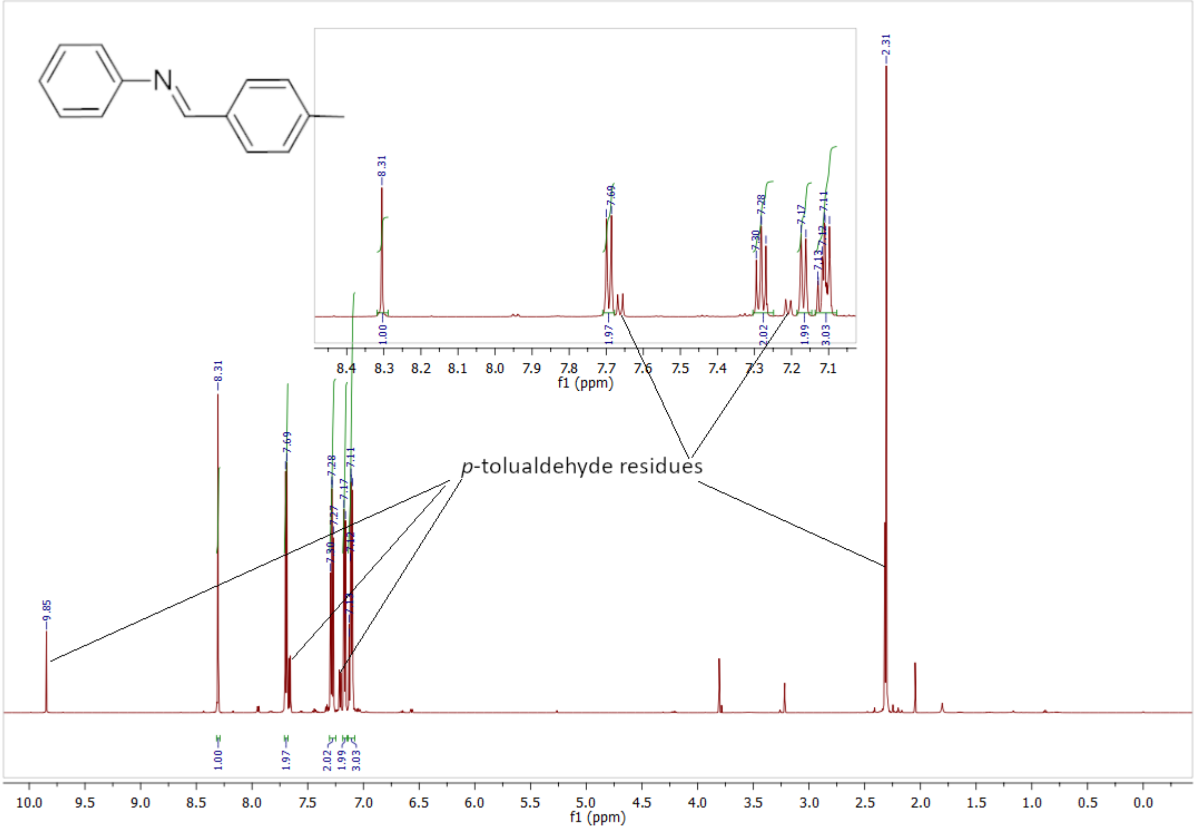

^{1}H NMR spectra of Schiff base **15a**

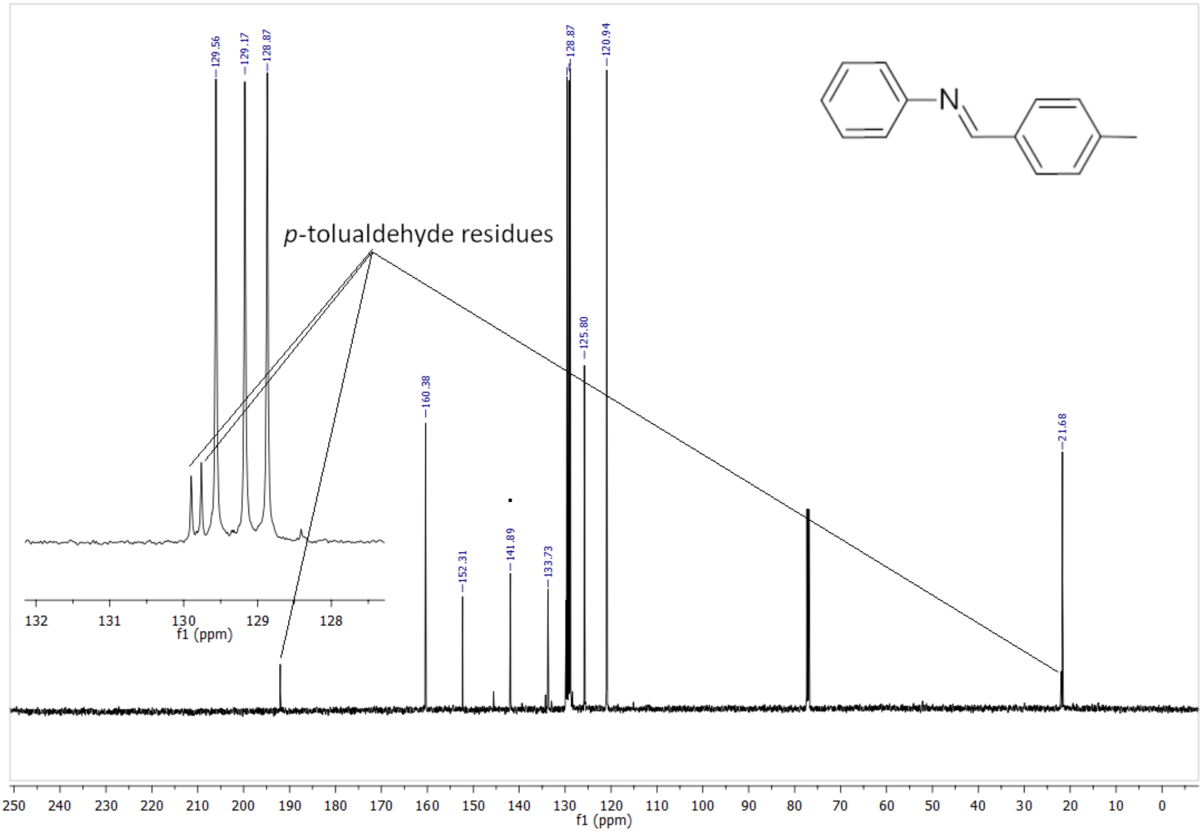

^{13}C NMR spectra of Schiff base **15a**

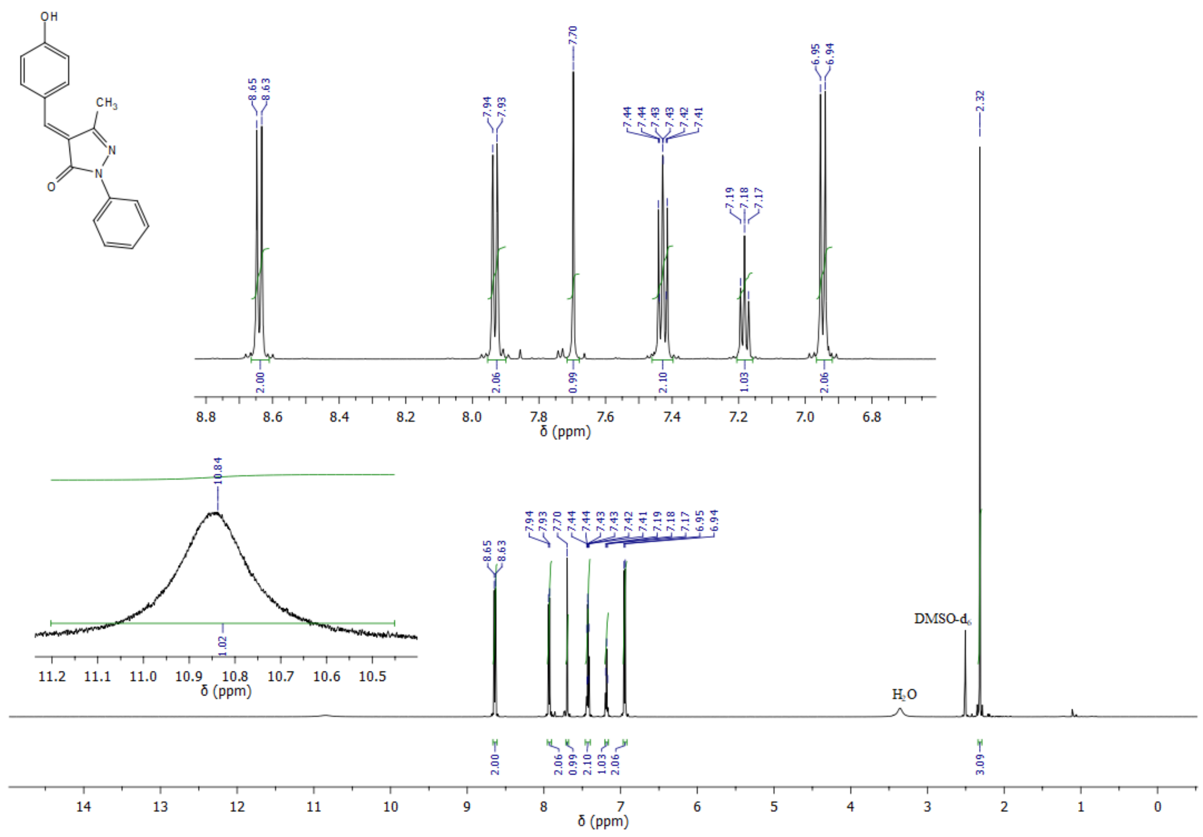

(4*E*)-4-[(4-hydroxyphenyl)methylidene]-5-methyl-2-phenyl-2,4-dihydro-3*H*-pyrazol-3-one **16a**

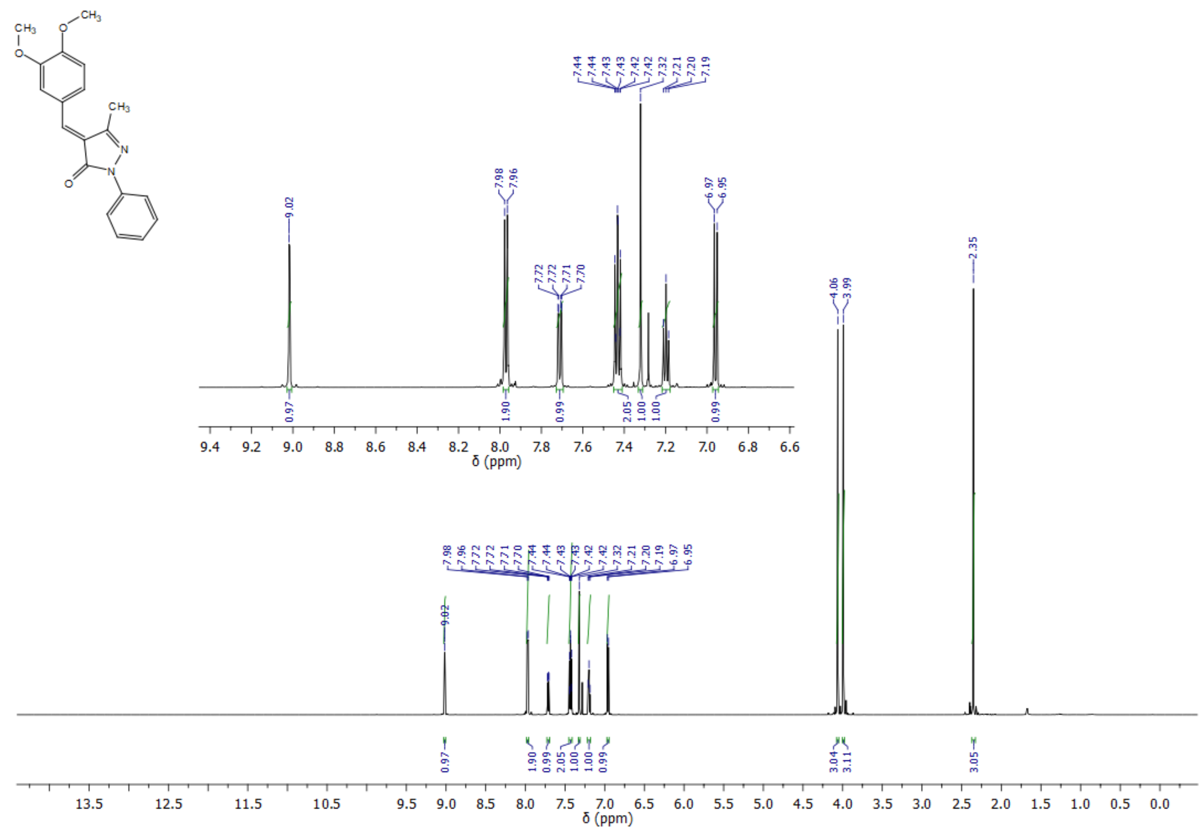

^{1}H NMR spectra of (4*E*)-4-[(3,4-dimethoxyphenyl)methylidene]-5-methyl-2-phenyl-2,4-dihydro-3*H*-pyrazol-3-one **16b**

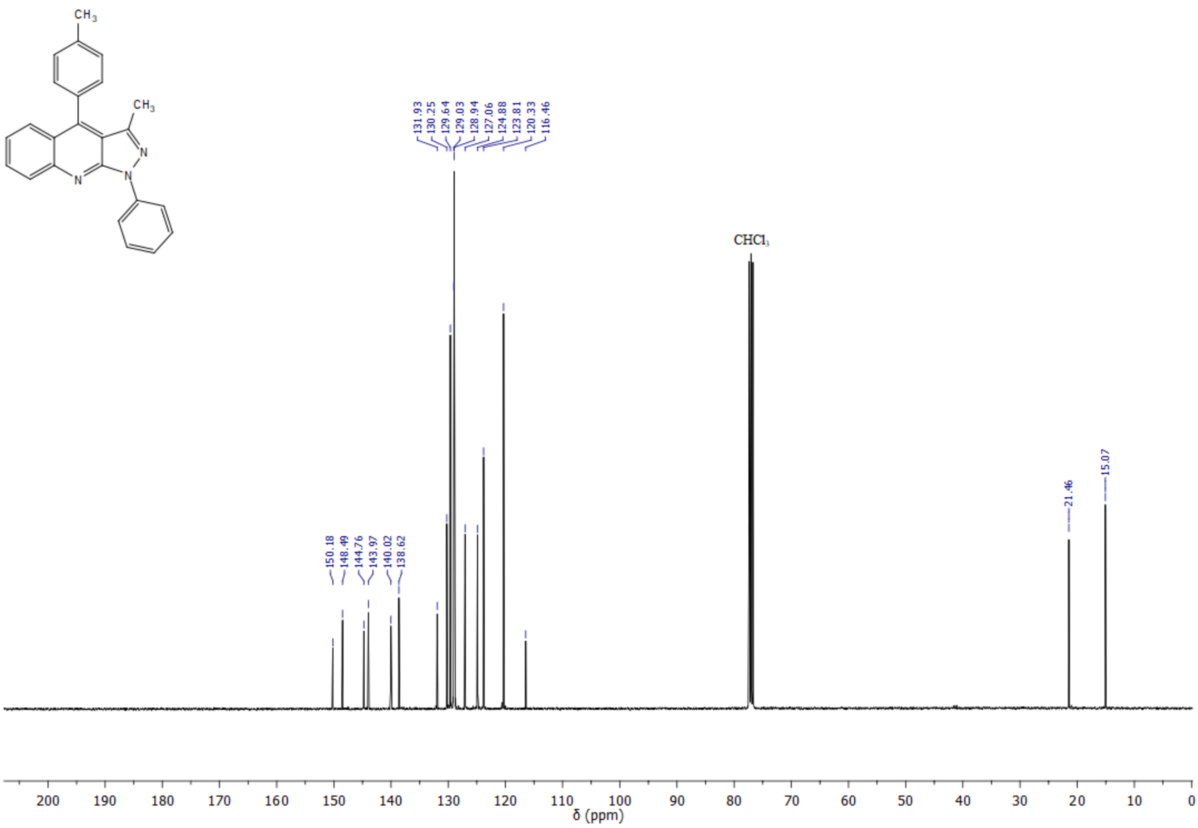

^{13}C NMR spectra of 4-(4-Methylphenyl)-3-methyl-1-phenyl-1*H*-pyrazolo[3,4-*b*]quinoline **7a**

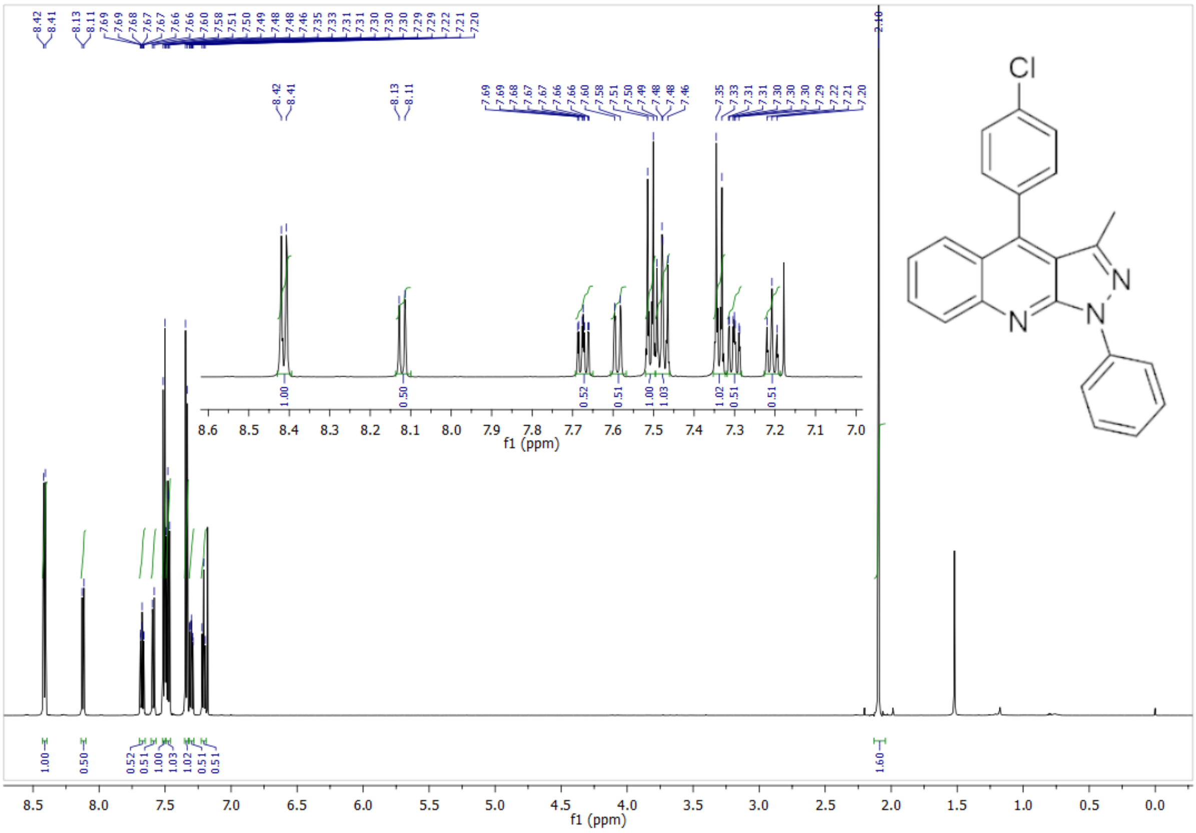

^{1}H NMR spectra of 4-(4-Chlorophenyl)-3-methyl-1-phenyl-1*H*-pyrazolo[3,4-*b*]quinoline **7b**

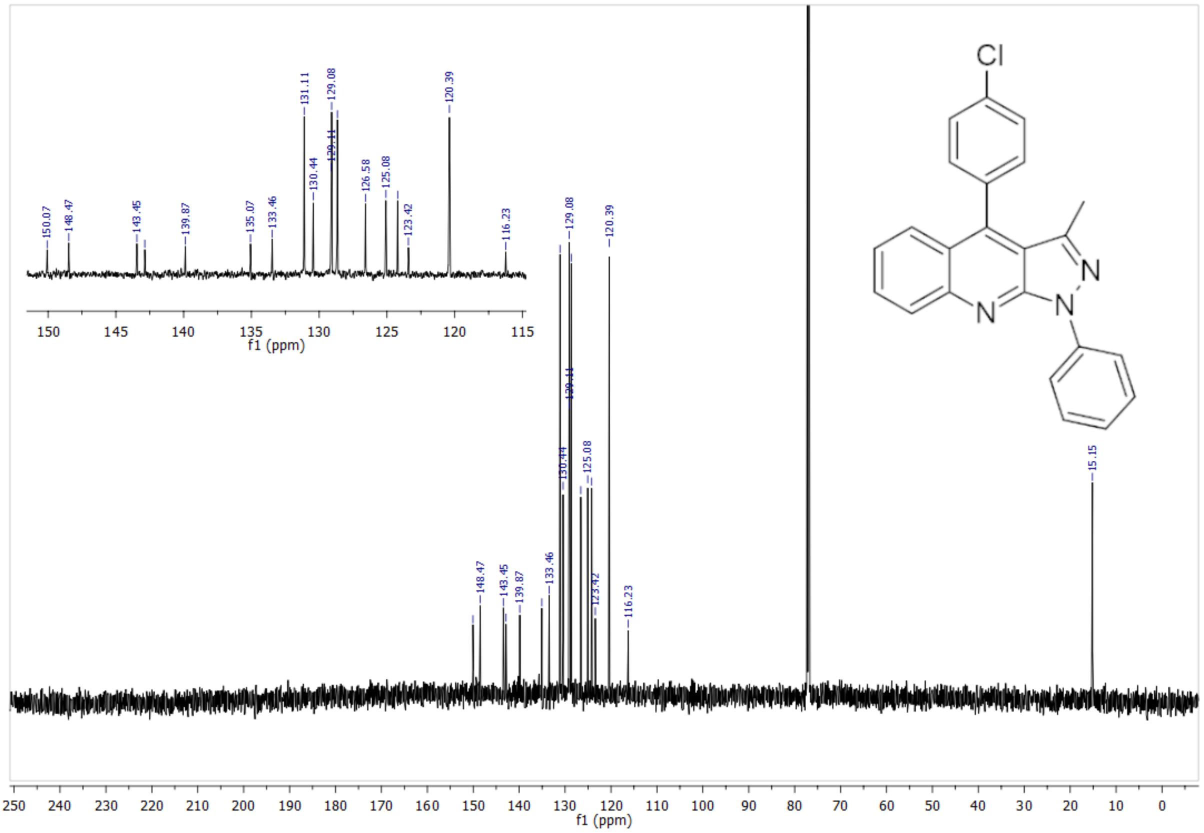

^{13}C NMR spectra of 4-(4-Chlorophenyl)-3-methyl-1-phenyl-1*H*-pyrazolo[3,4-*b*]quinoline **7a**

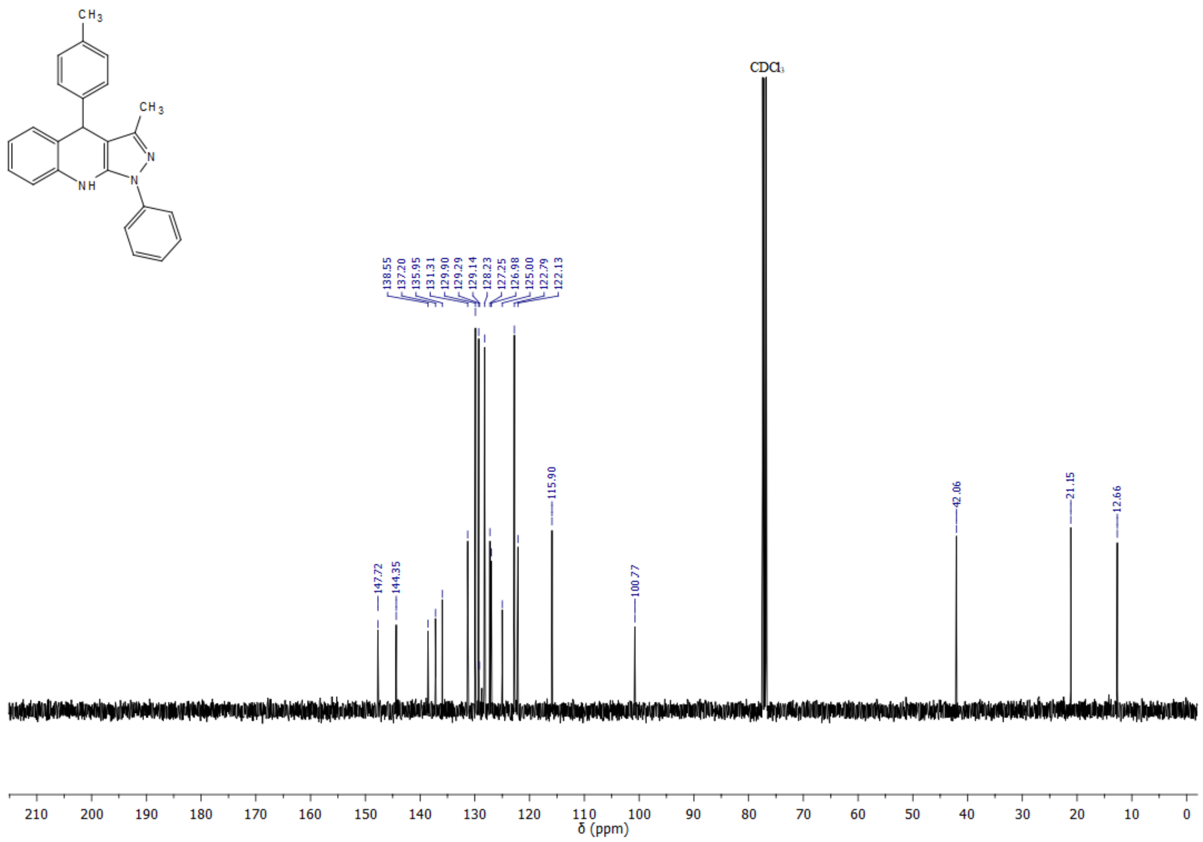

^{13}C NMR spectra of 4-(4-Methylphenyl)-4,9-dihydro-3-methyl-1-phenyl-1*H*-pyrazolo[3,4-*b*]quinoline **13a**

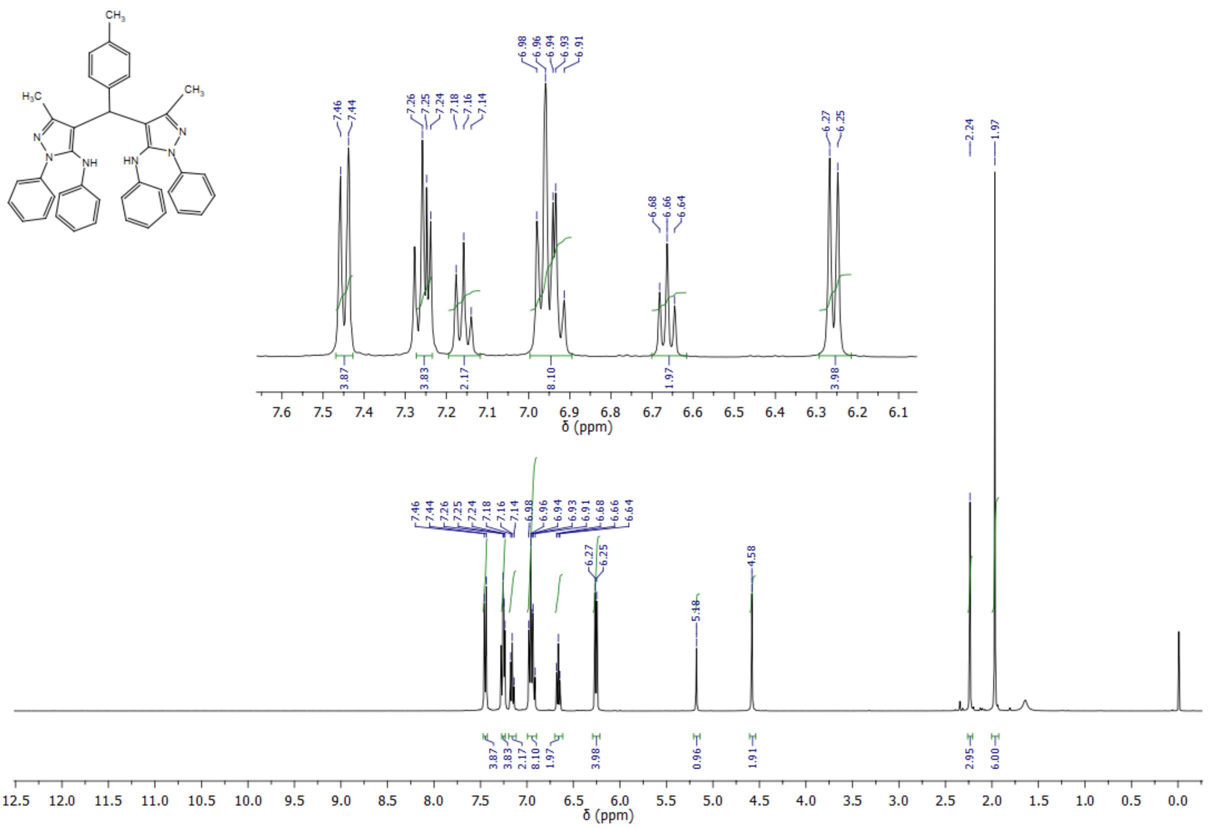

^{1}H NMR spectra of 4,4’-(4-Metylphenylmethylene)-bis-[3-methyl-*N*,1-diphenyl-1*H*-pyrazol-5-amine] **22a**

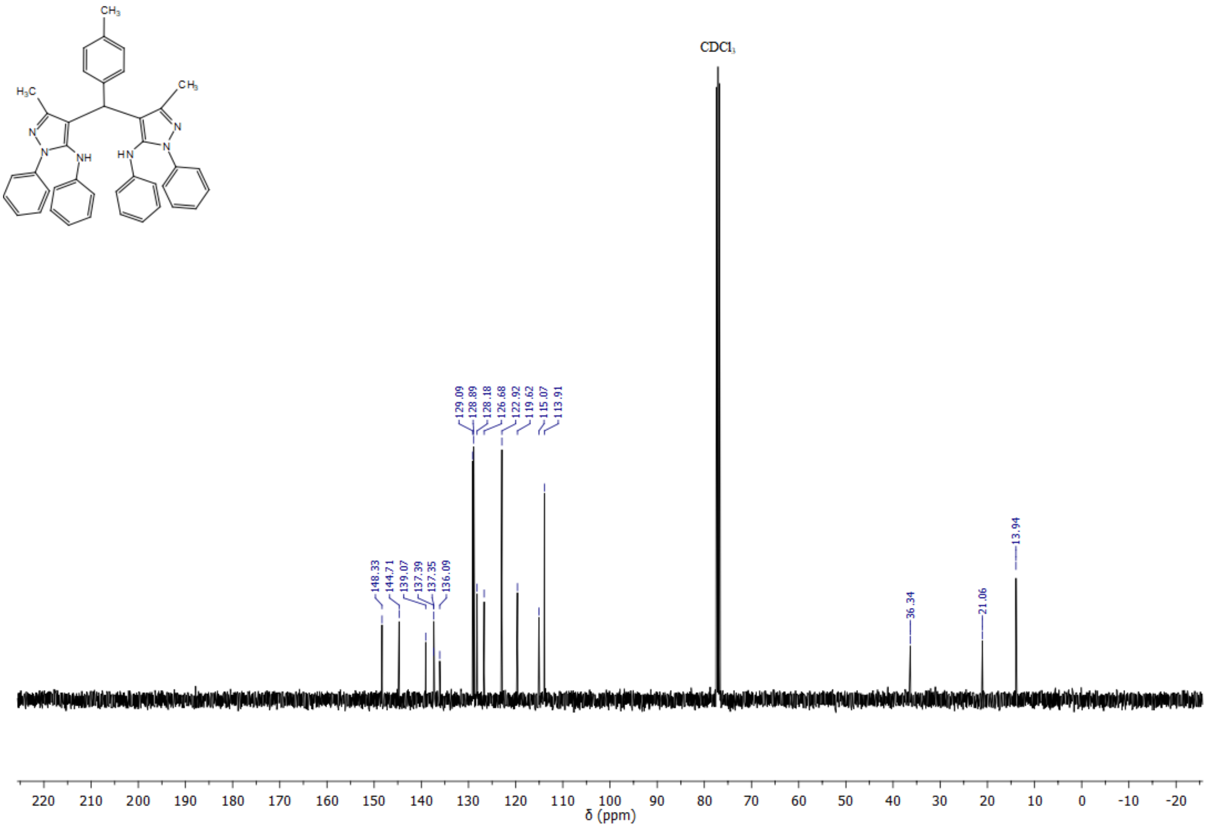

^{13}C NMR spectra of 4,4'-(4-Metylphenylmethylene)-bis-[3-methyl-*N*,1-diphenyl-1*H*-pyrazol-5-amine] **22a**

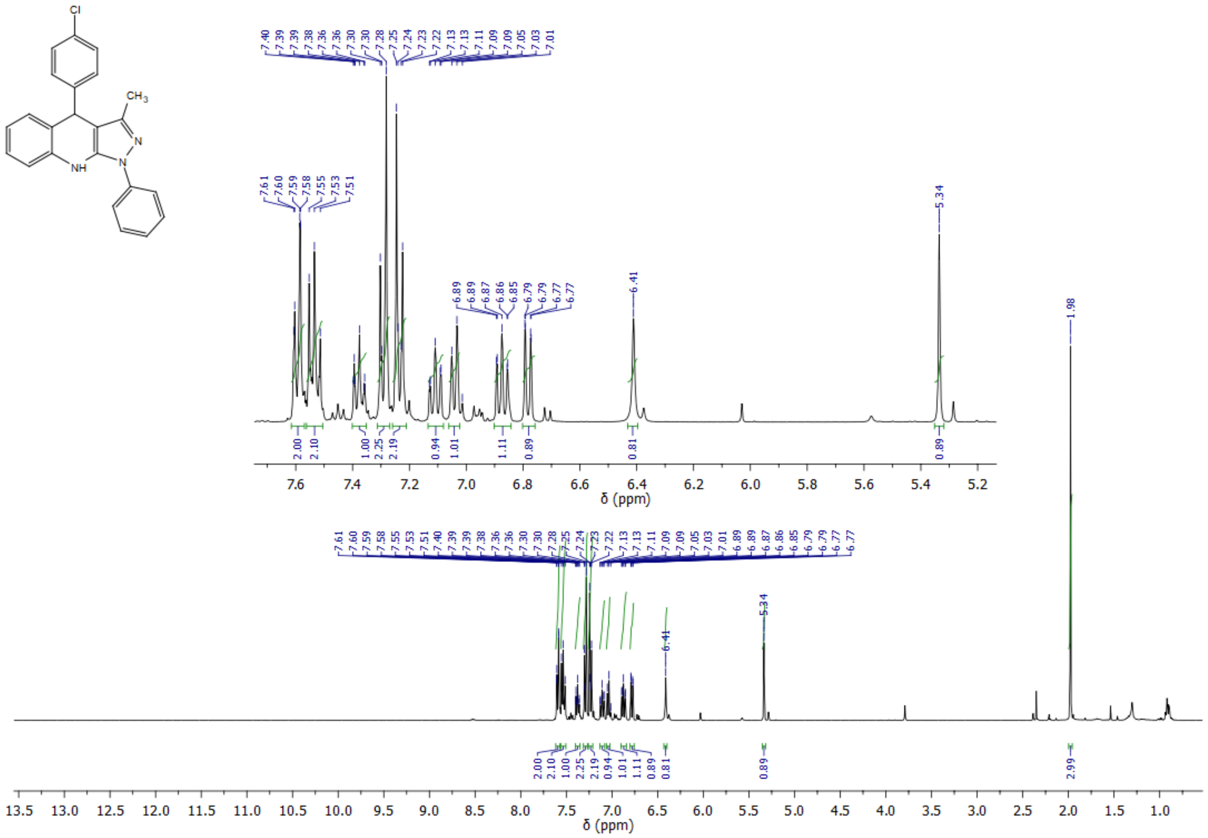

^{1}H NMR spectra of 4-(*p*-Chlorophenyl)-4,9-dihydro-1-phenyl-3-methyl-1*H*-pyrazolo[3,4-*b*]quinoline **13b**

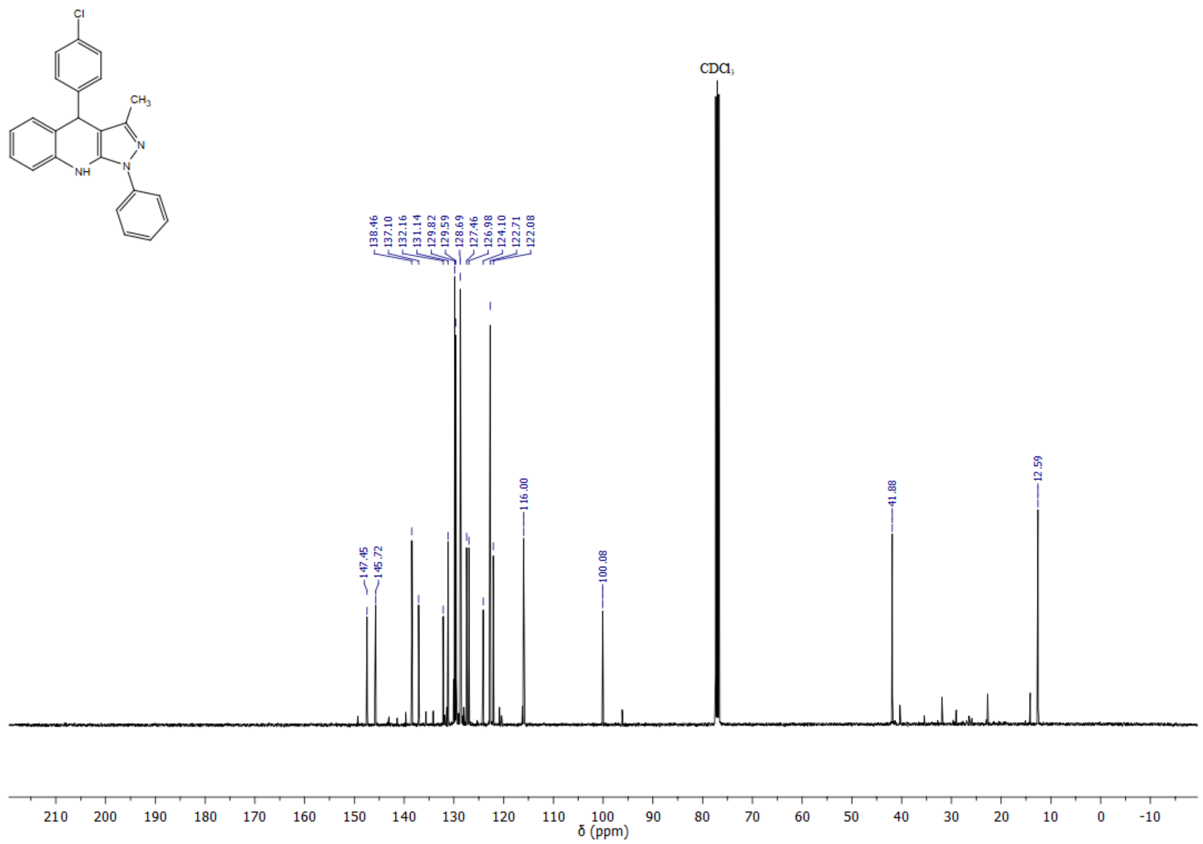

^{13}C NMR spectra of 4-(*p*-Chlorophenyl)-4,9-dihydro-1-phenyl-3-methyl-1*H*-pyrazolo[3,4-*b*]quinoline **13b**

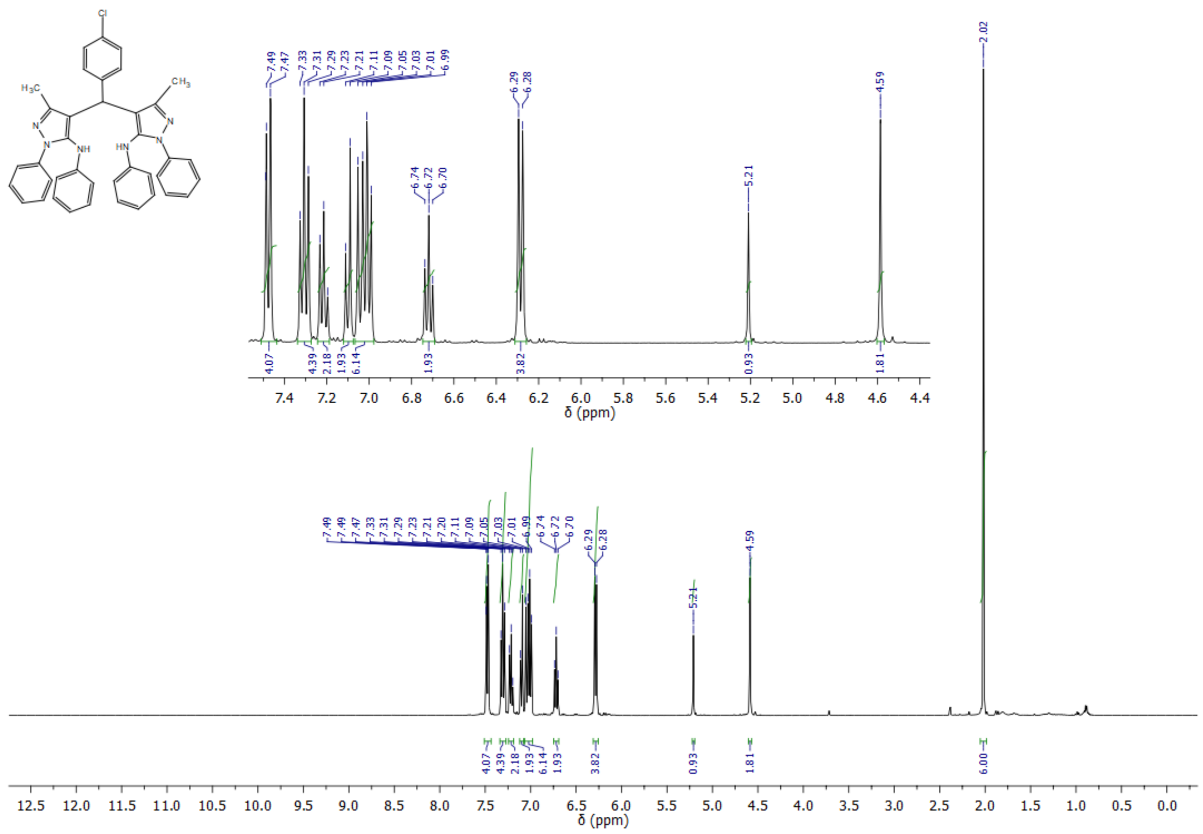

^{1}H NMR spectra of 4,4’-(4-Chlorophenylmethylene)-bis-[3-methyl-*N*,1-diphenyl-1*H*-pyrazol-5-amine)] **22b**

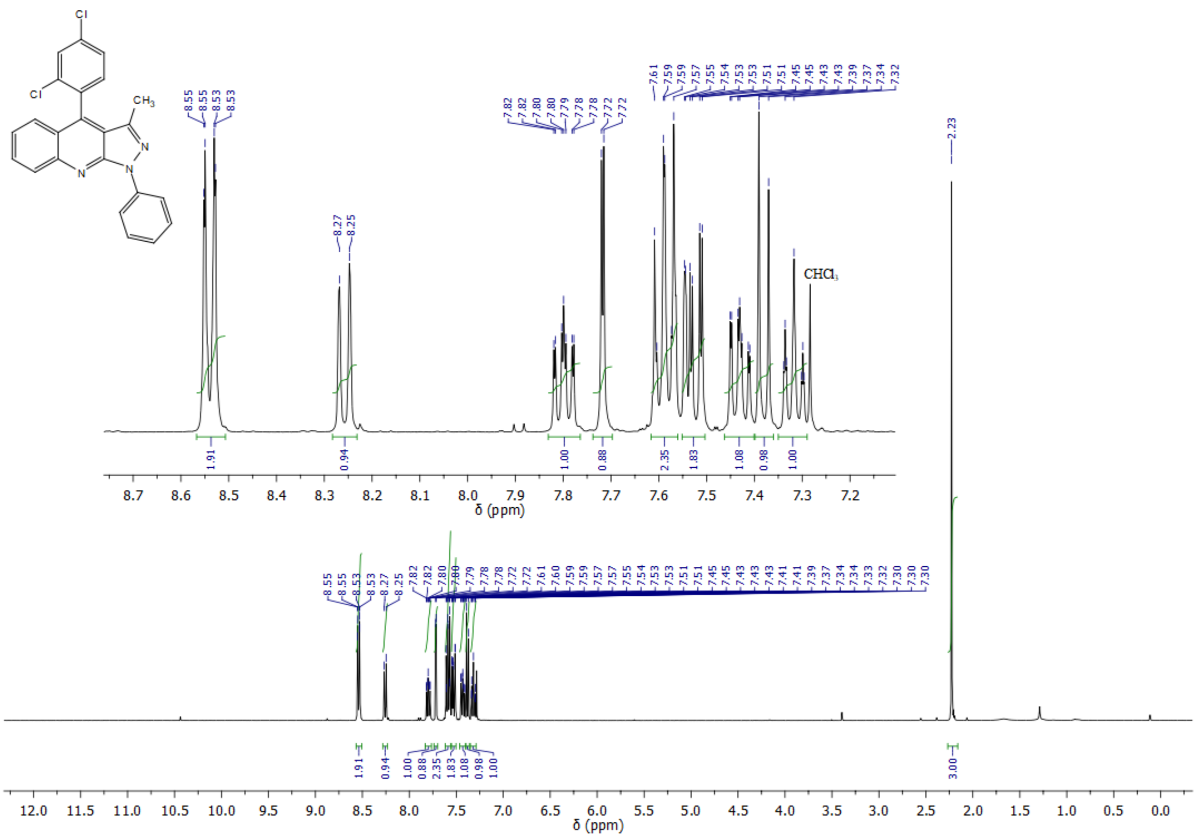

^{1}H NMR spectra of 4-(2,4-Dichlorophenyl)-1-phenyl-3-methyl-1*H*-pyrazolo[3,4-*b*]quinoline **7e**

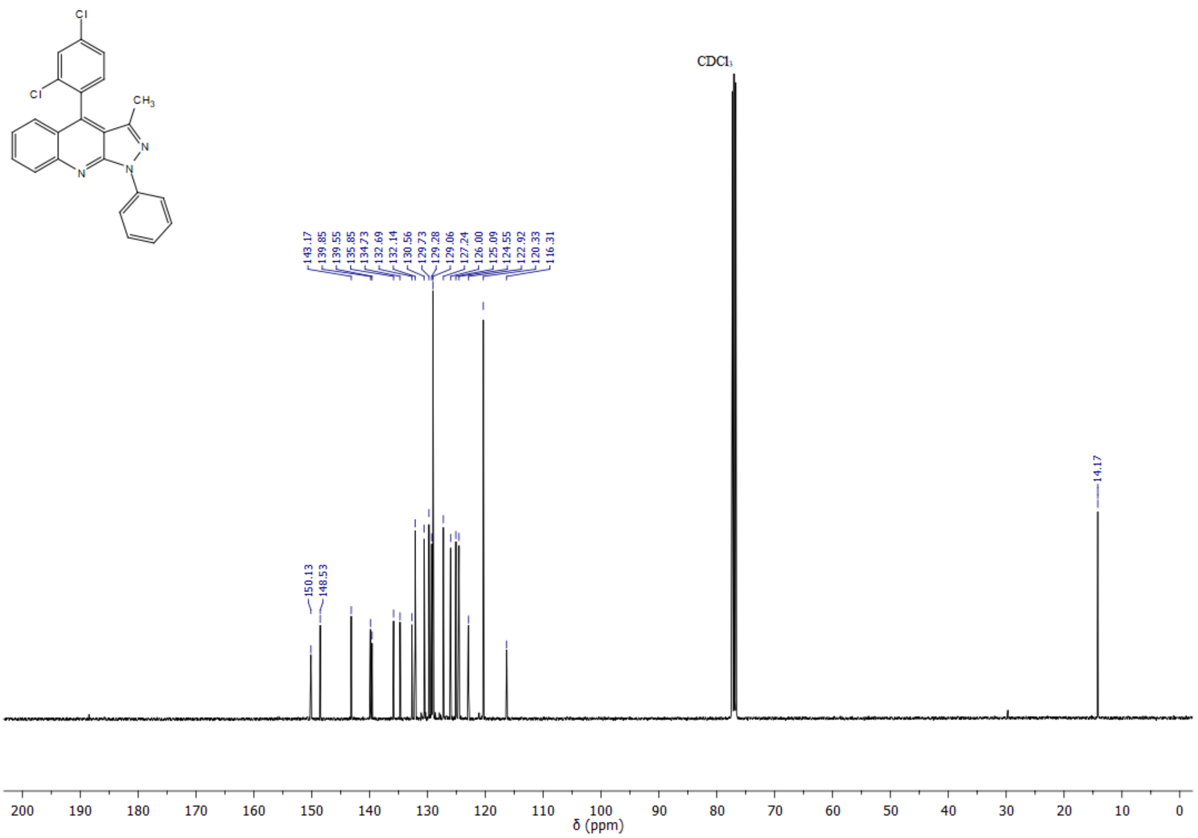

^{13}C NMR spectra of 4-(2,4-Dichlorophenyl)-1-phenyl-3-methyl-1*H*-pyrazolo[3,4-*b*]quinoline **7e**

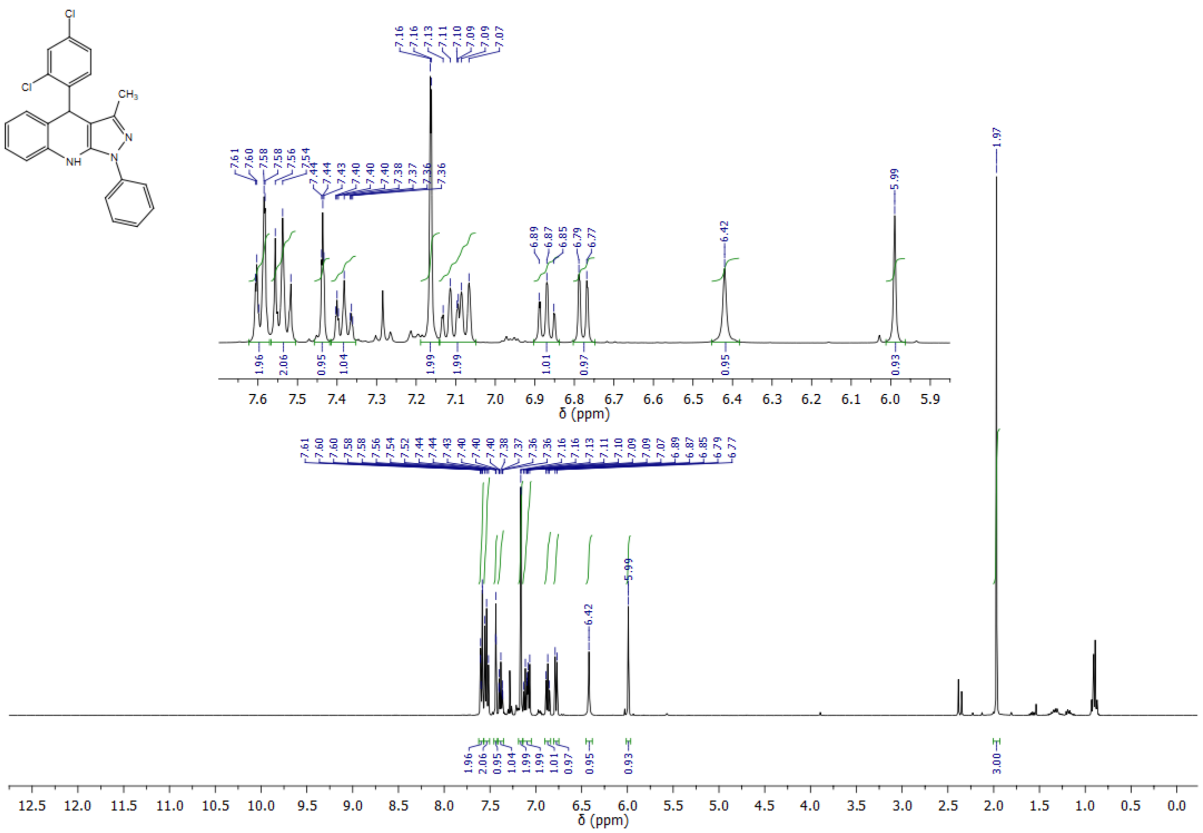

^{1}H NMR spectra of 4-(*p*-2,4-Dichlorophenyl)-4,9-dihydro-1-phenyl-3-methyl-1*H*-pyrazolo[3,4-*b*]quinoline **13e**

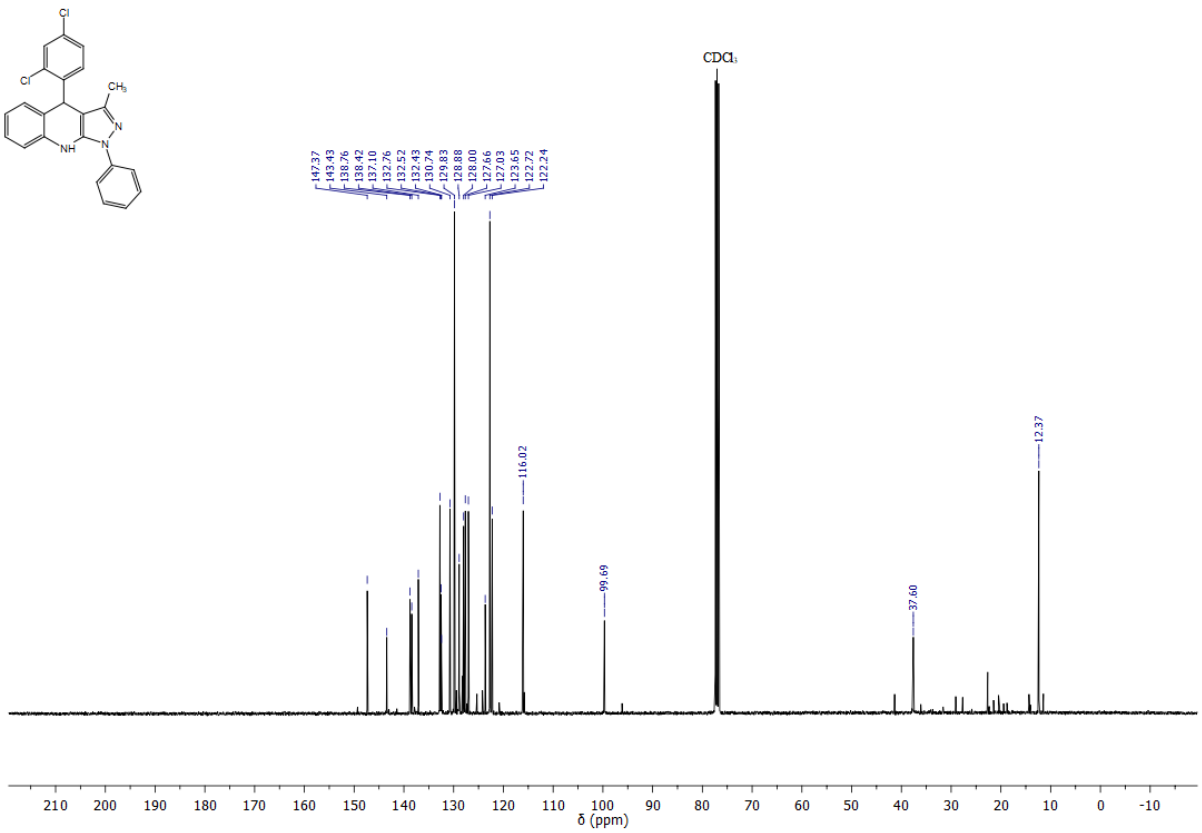

^{13}C NMR spectra of 4-(*p*-2,4-Dichlorophenyl)-4,9-dihydro-1-phenyl-3-methyl-1*H*-pyrazolo[3,4-*b*]quinoline **13e**

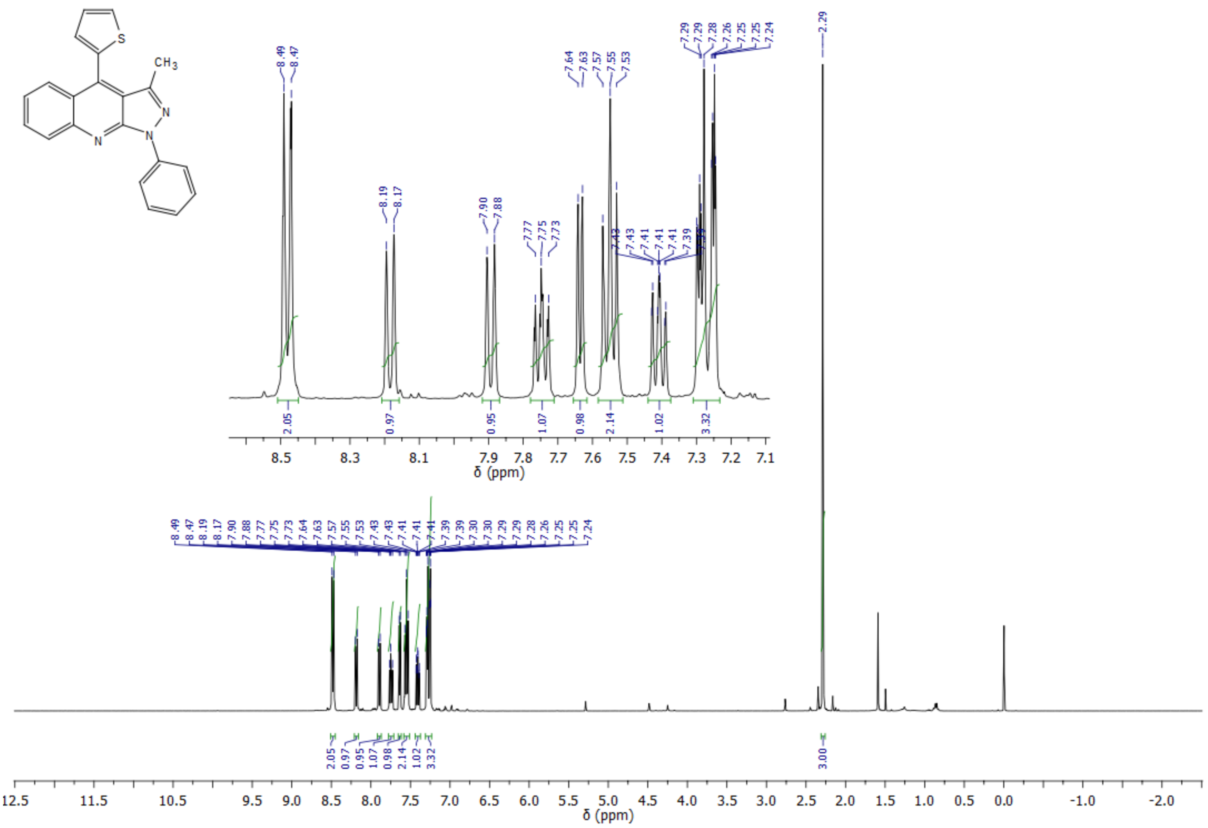

^{1}H NMR spectra of 4-(Thienyl-2-ylo)-3-methyl-1-phenyl-1*H*-pyrazolo[3,4-*b*]quinoline

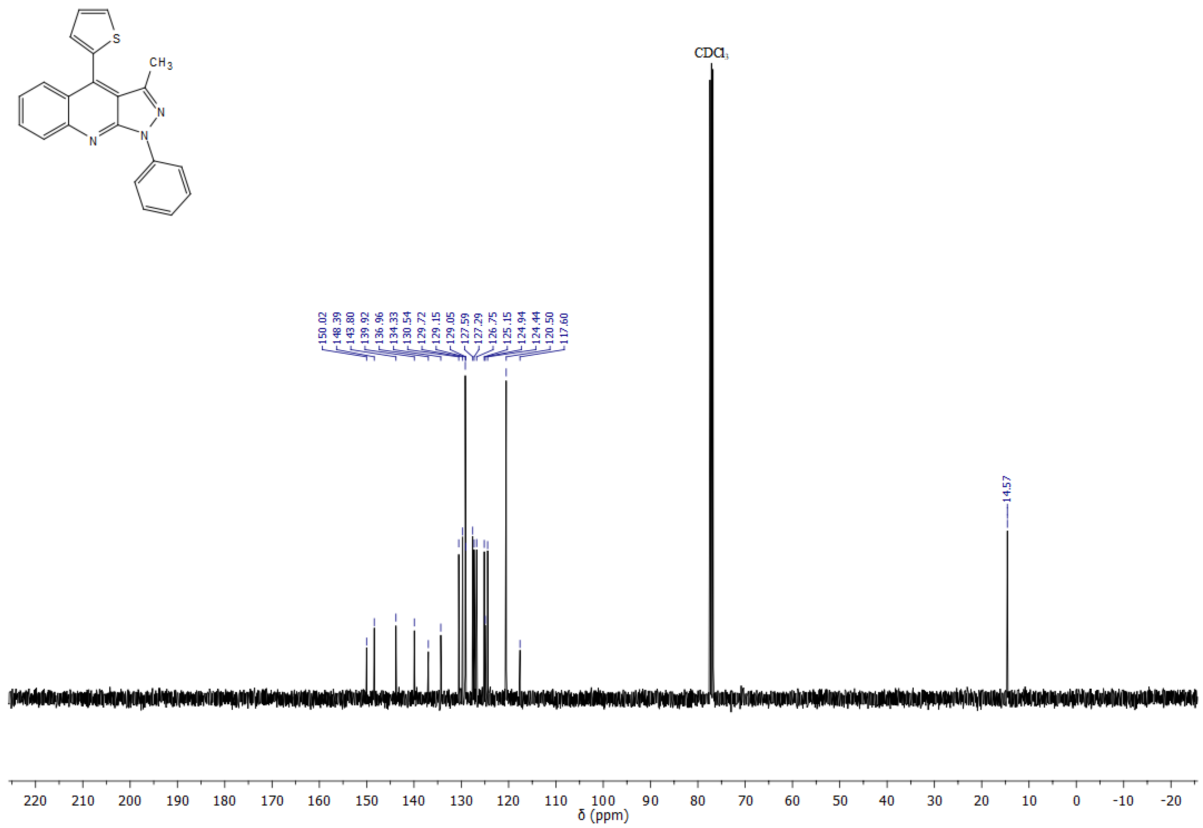

^{13}C NMR spectra of 4-(Thienyl-2-ylo)-3-methyl-1-phenyl-1*H*-pyrazolo[3,4-*b*]quinoline

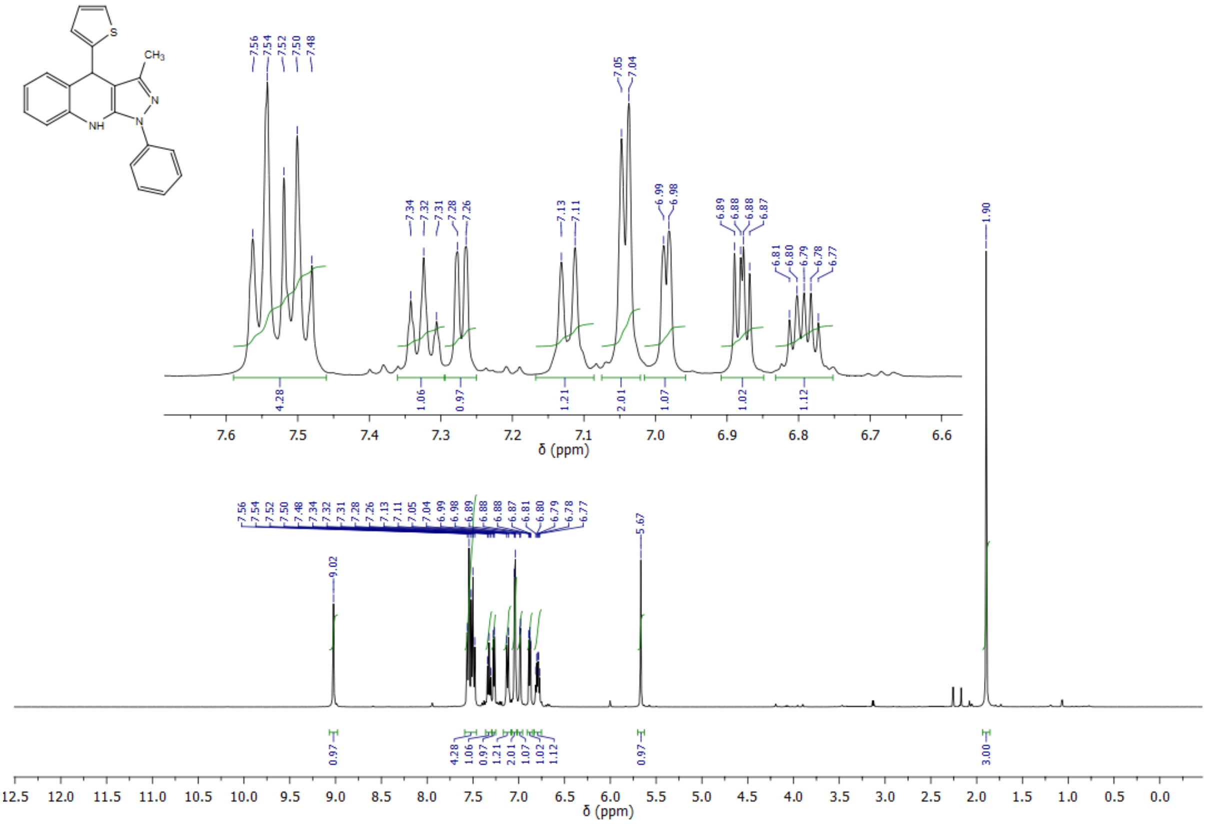

^{1}H NMR spectra of 4-(Thienyl-2-ylo)-4,9-dihydro-3-methyl-1-phenyl-1*H*-pirazolo[3,4-*b*]quinoline

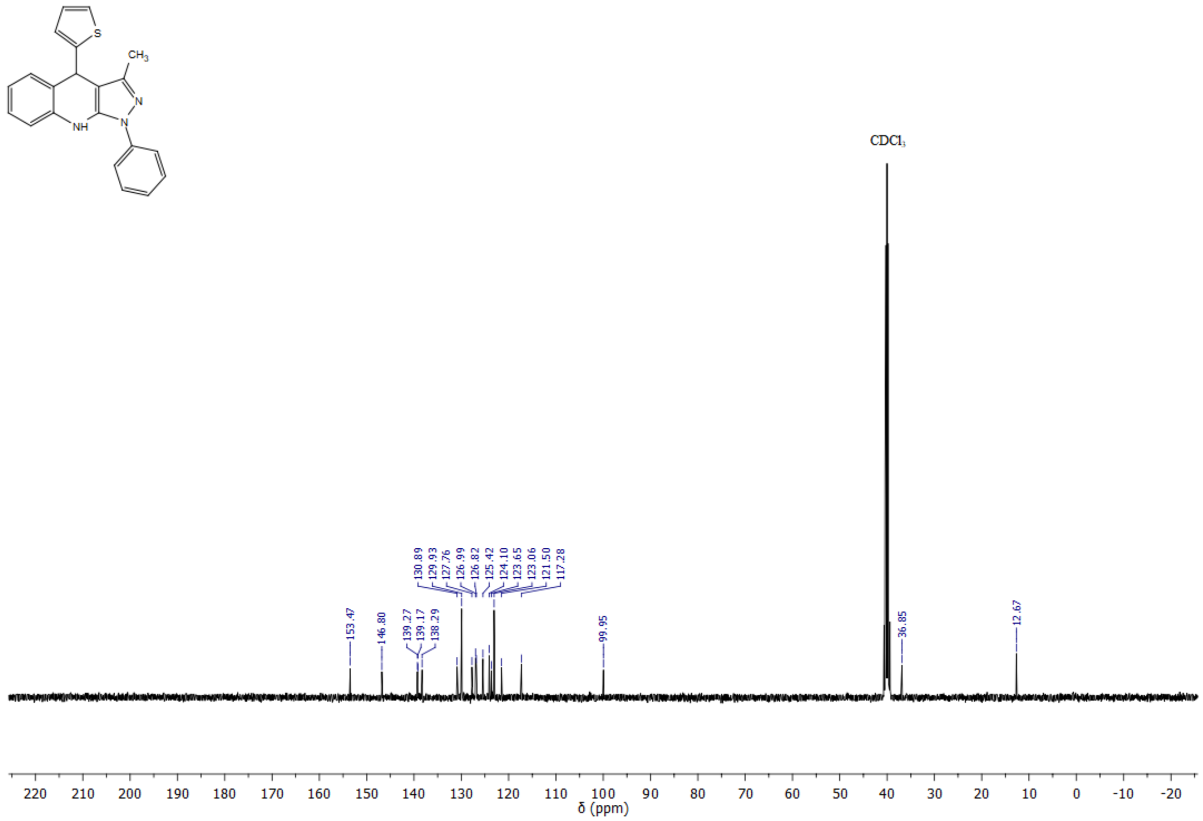

^{13}C NMR spectra of 4-(Thienyl-2-ylo)-4,9-dihydro-3-methyl-1-phenyl-1*H*-pirazolo[3,4-*b*]quinoline

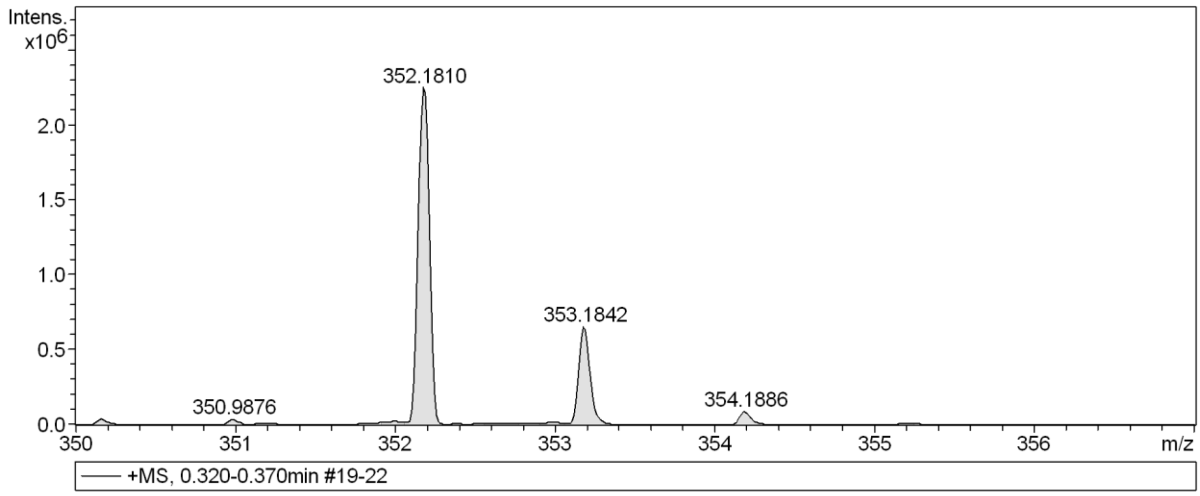

HRMS/ESI spectra of **13a**

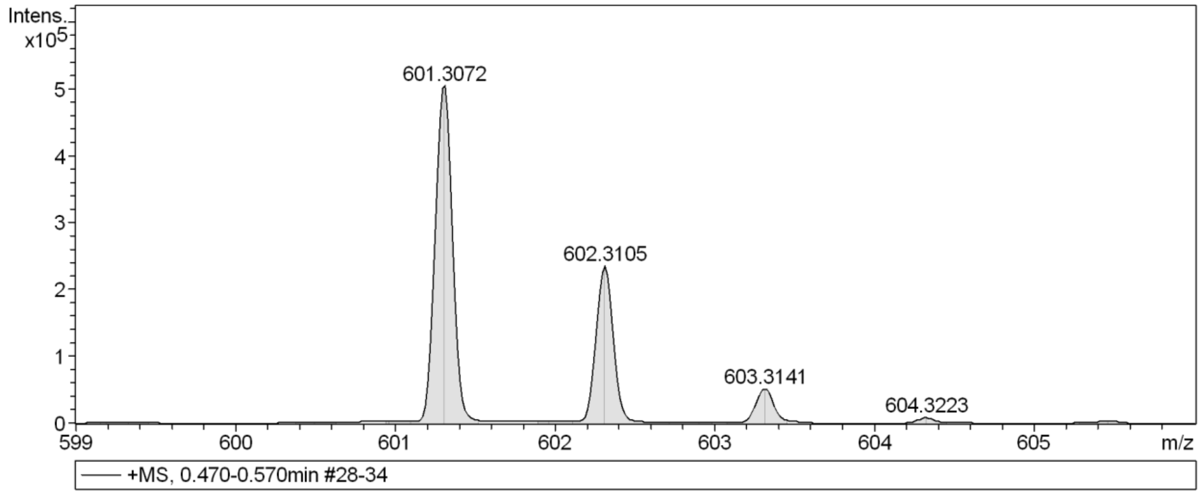

HRMS/ESI spectra of **21a**

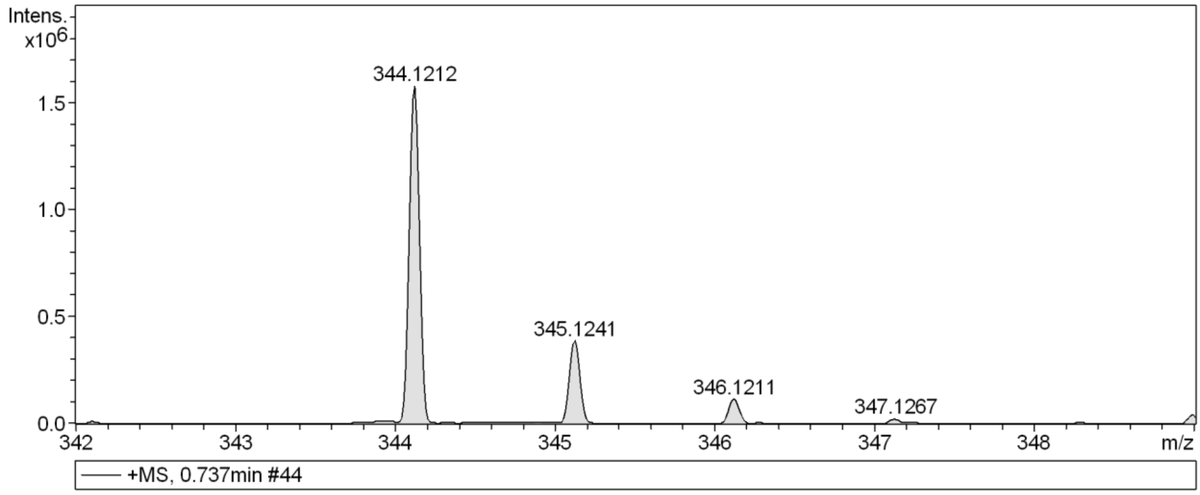

HRMS/ESI spectra of **13g**
